# Integrative genome-wide analysis of dopaminergic neuron-specific PARIS expression in *Drosophila* dissects recognition of multiple PPAR-γ associated gene regulation

**DOI:** 10.1038/s41598-021-00858-7

**Published:** 2021-11-02

**Authors:** Volkan Yazar, Sung-Ung Kang, Shinwon Ha, Valina L. Dawson, Ted M. Dawson

**Affiliations:** 1 Neuroregeneration and Stem Cell Programs, Institute for Cell Engineering, Johns Hopkins University School of Medicine, 733 North Broadway, Suite 711, Baltimore, MD 21205 USA; 2 Department of Neurology, Johns Hopkins University School of Medicine, Baltimore, USA; 3 Department of Physiology, Johns Hopkins University School of Medicine, Baltimore, USA; 4 Solomon H. Snyder Department of Neuroscience, Johns Hopkins University School of Medicine, Baltimore, USA; 5 Department of Pharmacology and Molecular Sciences, Johns Hopkins University School of Medicine, Baltimore, MD 21205 USA; 6 Adrienne Helis Malvin Medical Research Foundation, New Orleans, LA 70130-2685 USA; 7 Diana Helis Henry Medical Research Foundation, New Orleans, LA 70130-2685 USA

**Keywords:** Gene expression profiling, Computational biology and bioinformatics, Cell death in the nervous system, Diseases of the nervous system

## Abstract

The transcriptional repressor called parkin interacting substrate (PARIS; ZNF746) was initially identified as a novel co-substrate of parkin and PINK1 that leads to Parkinson’s disease (PD) by disrupting mitochondrial biogenesis through peroxisome proliferator-activated receptor gamma (PPARγ) coactivator -1α (PGC-1α) suppression. Since its initial discovery, growing evidence has linked PARIS to defective mitochondrial biogenesis observed in PD pathogenesis. Yet, dopaminergic (DA) neuron-specific mechanistic underpinnings and genome-wide PARIS binding landscape has not been explored. We employed conditional translating ribosome affinity purification (TRAP) followed by RNA sequencing (TRAP-seq) for transcriptome profiling of DA neurons in transgenic *Drosophila* lines expressing human PARIS wild type (WT) or mutant (C571A). We also generated genome-wide maps of PARIS occupancy using ChIP-seq in human SH-SY5Y cells. The results demonstrated that PPARγ functions as a master regulator of PARIS-induced molecular changes at the transcriptome level, confirming that PARIS acts primarily on PGC-1α to lead to neurodegeneration in PD. Moreover, we identified that PARIS actively modulates expression of PPARγ target genes by physically binding to the promoter regions. Together, our work revealed how PARIS drives adverse effects on modulation of PPAR-γ associated gene clusters in DA neurons.

## Introduction

As the second most common neurodegenerative disorder worldwide with a prevalence as high as 572 people per 100,000 in the US^1^, Parkinson’s disease is now referred to as a “silent epidemic” of our times. This brain disorder is pathologically characterized by selective loss of dopamine neurons in the substantia nigra leading to motor system dysfunctions including bradykinesia, resting tremor, rigidity and muscular rigidity^2–4^. In recent years, our understanding of genetic predisposition to PD has advanced vastly, including causative mutations in the genes encoding α-synuclein, LRRK2, parkin, PINK1, and DJ-1 just to name a few^5^. Accumulating molecular evidence suggests a general involvement of mitochondrial dysfunction in the underlying molecular mechanism of PD but the mechanistic underpinnings of this link are poorly defined. In this respect, the transcriptional repressor PARIS (ZNF746) was identified by our group in 2011^6^ among several other co-substrates of PINK1 and parkin as a promising candidate to shed light on possible contributions of a defective mitochondrial biogenesis to PD pathogenesis. In the same paper we also demonstrated that accumulating PARIS acts on PGC-1α, the master regulator of mitochondrial biogenesis, along the NRF1/2-TFAM axis to intervene in mitochondrial biogenesis, and that the transcriptionally inactive PARIS mutant (C571A) lacks repression capacity to induce the phenotype. Recently two variants in PARIS were identified in early onset Parkinson’s disease in Chinese providing a genetic link of PARIS to PD^7^.

Originally discovered as a chief regulatory protein of glucose and lipid metabolism and cell differentiation, the peroxisome proliferator-activated receptor gamma (PPARγ) is a ligand-activated transcription factor of the nuclear hormone receptor superfamily^8^. It is also well-established that PPARγ is associated with key molecular networks in cells, including redox balance, fatty acid oxidation, and mitochondrial function, as well as neuronal (out)growth, differentiation, and polarity^9–12^. To achieve this versatility PPARγ interacts with a range of additional molecules required for both transcriptional co-activation and structural changes that promote its dimerization for DNA binding. Indeed, some of these PPARγ activators have already been suggested as potential targets for the treatment of several neurodegenerative disorders including PD, Alzheimer’s disease, Huntington’s disease and amyotrophic lateral sclerosis (ALS)^13^. Overall, the current knowledge of mechanisms underlying the beneficial effects of PPARγ agonists and PGC-1α in models of PD has been discussed elsewhere^14^.

Here, we employed conditional translating ribosome affinity purification (TRAP)^15^ followed by RNA sequencing (TRAP-seq) for transcriptome profiling of dopaminergic (DA) neurons in transgenic *Drosophila* lines expressing human PARIS WT or mutant (C571A). The results revealed that we successfully isolated DA neuron-specific mRNAs from *Drosophila*. As a complementary approach, we performed a global, model-based analysis of PARIS genomic occupancy using chromatin immune-precipitation followed by sequencing (ChIP-seq) on SH-SY5Y (in-house dataset) and 293 T (public dataset) human cell lines. By combining these two approaches in a single study to measure the regulatory capacity of PARIS, we identified a set of direct target genes that are also differentially expressed by PARIS. Based on these findings, we showed using both approaches separately and combined that PPARγ acts as a potential master regulator of transcriptomic changes induced by PARIS in the clusters of *Drosophila* DA neurons. Also, we revealed that PARIS directly modulates expression of PPARγ target genes by physically binding to the promoter regions. And finally, we observed a PARIS binding motif at the promoter proximal site of PPARγ, implying a direct regulatory effect of PARIS on PPARγ expression profile. Together, our studies shed light on the molecular basis that gives rise to dopaminergic neurotoxicity induced by PARIS accumulation in PD.

## Methods

### Maintenance of hPARIS-expressed TRAP Fly, and Sequencing library generation for TRAP-seq

Human PARIS and C571A constructs cloned and expressed in pUAST plasmid (DGRC) were microinjected into w1118 embryos (The BestGene, Inc)^5^. Transgenic *dTH-Gal4* lines were obtained from the Bloomington Drosophila Stock Center (BDSC). All experimental maintenance and crosses between transgenic lines were made at 25 °C. Drosophila melanogaster fly stocks were handled using standard protocols, maintained in a 12 h light/dark cycle and fed Drosophila standard diet consisting of cornmeal, agar, yeast, sucrose, and dextrose. For the TRAP experiments, both male and female flies were used. In each experiment, the *dTH-Gal4* heterozygous flies were used as control. Prior to the TRAP, no *Gal4* titration was performed experimentally. To match the male/female ratio and developmental stage between control and experimental groups, we collected equal numbers of males and females two days after eclosion. For the TRAP analysis of fly DA neurons, translated mRNAs in DA neurons were purified from *dTH-GAL4/UAS-GFP::RpL10A* with and without *hPARIS WT* or *mutant (C571A)* as described previously^15^. The libraries were prepared from three independent biological samples from 500 (control and hPARIS mutant lines)—1500 (hPARIS WT line) of this mixture of male and female heads, whereas a sequencing library using the total mRNA from whole head was prepared for one biological sample to provide quality-assurance of DA neuron-specific translatome. Libraries for RNA sequencing were prepared with the TruSeq RNA Sample preparation v2 kit (Illumina) and sequenced on an Illumina HiSeq 2000 sequencer.

### Real-time Quantitative RT-PCR (qRT-PCR)

Purified total RNAs aliquoted from PARIS-expressed TRAP Fly RNA extraction steps were used for cDNA synthesis using a First-strand cDNA synthesis kit (Invitrogen). Aliquots of cDNA were used as templates for real-time qRT-PCR procedure. Relative quantities of mRNA expression were analyzed using a SYBR green PCR kit (Qiagen) according to the manufacturer’s instruction. Statistical significance was determined by unpaired student’s *t* test using GraphPad prism software. Asterisks denote statistical significance (****p < 0.0001) as compared to controls.

The oligonucleotide sequences are listed below:

**Table:** 

Name of application	Gene name	Sequence
Fly_gene set	fly_Appl_F	CGTCTACTTCACGCTCTCCT
	fly_Appl_R	GACCTCGATGAAGCCCTGG
	fly_Porin2_F	GGTAACCAAGGGAGCCGG
	fly_Porin2_R	CAGTTTCTGTTCATAGCCCAGG
	fly_Dark_F	AGCAGACTTCAACCTCCACC
	fly_Dark_R	CTTCTCCAGAACCAGGTTGC
	Fly_Nrv2_F	CATTTGGGTATCGTGCGAGG
	Fly_Nrv2_R	TCAAGTAGCCCTCGGAGTTC

### Immunoblot

Total lysates or GFP + immunoprecipitated samples from 500 to 700 of a mixture of male and female heads were separated by 8–16% SDS-PAGE and transferred to nitrocellulose membrane (0.45 μm)^16^. 5% Difco skim milk (BD Bioscience) in PBST (0.05% Tween 20) was incubated for blocking, and the membranes were applied with specific antibodies as described in the figures. After incubation with horseradish peroxidase-conjugated secondary anti-mouse IgG (Amersham Bioscience), the antigen–antibody was detected in X-ray film (AGFA) by an ECL method (Thermo Scientific)^16^. The original membranes were cut prior to hybridization with antibodies during blotting. Primary antibodies used include the following: mouse monoclonal anti-GFP (N86/38, NeuroMab) and anti-beta-Actin HRP conjugate (13E5, Cell Signaling).

### PARIS ChIP-sequencing in SH-SY5Y human neuroblastoma cells

Human SH-SY5Y neuroblastoma cells were purchased from American Type Culture Collection (ATCC, Manassas, VA) and cultured in Dulbecco’s modified Eagle’s medium (DMEM) supplemented with 10% heat-inactivated fetal bovine serum (FBS) and 1% penicillin/streptomycin (Gibco-Invitrogen, Carlsbad, CA). For transient transfection, cells were transfected with indicated amounts of either flag-PARIS or flag using Fugene HD (Promega, Madison, WI) according to manufacturer’s instructions. After 48 h incubation, SH-SY5Y cells were cross-linked with 1% formaldehyde for 10 min at room temperature, and 125 mM glycine were then added to inactivate the formaldehyde. CHIP-seq high sensitivity kit (ab185908, Abcam) was further used for ChIP-Seq library preparation according to manufacturer’s instructions. Libraries were sequenced on an Illumina HiSeq 2000 sequencer.

### ChIP-qPCR

ChIP-qPCR was performed using SH-SY5Y cells transfected with Flag-PARIS^17^. After 48 h incubation, cells were cross-linked with 1% formaldehyde for 10 min at room temperature. Nuclei were lysed and the chromatin was digested to 150–900 bp by micrococcal nuclease for 20 min at 37 °C followed by sonication with three sets of 20-s pulses. Chromatin was rotated overnight at 4 °C with 5 μg of either Flag antibody (Sigma-Aldrich), Rabbit IgG (Cell signaling) as a negative control, or histone antibody (Cell signaling) as a positive control. Antibody-DNA complexes were isolated by ChIP grade protein G-agarose beads, eluted from the beads, and digested by 40 μg of Proteinase K for 2 h at 65 °C, followed by spin column-based purification of the DNA^17^. Primers were designed based on PARIS-DNA binding regions identified.

The oligonucleotide sequences are listed below:

**Table:** 

Name of application	Gene name	Sequence
Human gene set	APAF1_Ch_F	TGGACGTGACTGCTCTATCC
	APAF1_Ch_R	ACTGGACACAAAGGGAGGAG
	VDAC_Ch_F	ATCCAAAGTCAGGCTCCGAA
	VDAC_Ch_R	GTCGACTTAGGCGGTAGAGG
	APP_Ch_F	GCGAAAAGAGGTTGGAGCAA
	APP_Ch_R	TCCCTAAAGCCAGTCCTTCG
	ATP1B1_ch1_F	TGGCCTGAGTCTCAATTGGT
	ATP1B1_ch2_R	CTTCCCTCTTCTTTGCAGGC
	ATP1B1_ch2_F	GAGGAGGGCAGCTGGAAG
	ATP1B1_ch2_R	GAAAGATCAGACGCGGCG

### PARIS DNA binding affinity assay

PARIS-motif binding activity was determined using EpiQuik General Protein-DNA Binding Assay Kit—Colorimetric (Epigentek, NY, USA) according to the manufacturer's instructions. The absorbance was measured at 450 nm using Varioskan LUX (ThermoFisher, MA, USA) and SkanIt Software. Based on bioinformatics analysis, biotin–labeled oligonucleotides were constructed (Integrated DNA Technologies, IA, USA) using the following sequences: “New motif” oligonucleotide sequence: > 5′-GCCGGTG**GGCGCGGAGCCG**GGGACACG-′3**.** “Old motif” oligonucleotide sequence: > 5′-GCCGGTG**TATTTTT**GGGACACG-′3.

### RNA-seq data analysis

The TRAP-seq dataset composed of a total of 10 fastq files representing 4 different treatment groups was processed using the “alternate” differential expression workflow, a modified version of the “new” Tuxedo pipeline at http://ccb.jhu.edu/software/stringtie/index.shtml?t=manual#deseq. Briefly, after quality control (FASTQC v0.11.6) read alignment was performed using HISAT2 v2.1.0^18^, default parameters with “min-intronlen 74” and “max-intronlen 75,000”, Ensemble BDGP6.28 fly genome and the corresponding transcriptome annotation. After multi-mapped reads were identified exclusively specific for rRNA genes, StringTie v2.0’s by-default multi-mapping correction was enabled during transcript abundance estimations and generation of read coverage tables not to compromise the dataset by discarding multi-mapped reads, keeping the sample size (i.e. per-replicate-read-number) above the accepted threshold. StringTie output was directly analyzed for transcript-level differential expression (DE) using the R^19^ package Ballgown v2.16.0 while the same output was preprocessed using the author-supplied script “prepDE.py” at the website above for format conversion. These preprocessed files were fed into the R package DESeq2 v1.24.0^20^ for gene-level DE analysis, which ultimately generated the input for the rest of the downstream analysis presented here. The R package NOISeq(sim) v2.28.0 was used for DE analysis of pairwise comparisons with no replicates. Differentially expressed genes (DEGs) were identified with an adjusted *p* value (i.e. *q* value; BH-corrected) cutoff of less than 5%, which also applies to the rest of the workflow. FPKM-normalized reads were used to generate heatmaps with the R package pheatmap v1.0.12. Venn diagrams were generated using the “venn()” function in the R package gplots v3.0.1.1. Human orthologs of fly DEGs were identified using the most up-to-date version of HGNC tool, HCOP v02.28.2020, and downloaded in bulk for processing in python v3.6. These orthologs were evaluated in biological context using functional annotation tool of the Database for Annotation, Visualization, and Integrated Discovery (DAVID v6.8) with the human genome being the background^21–23^. More importantly, these orthologs, together with their corresponding fold changes and individual *p* values, were uploaded to the Ingenuity Pathway Analysis (IPA v2.4) server for in-depth knowledge analysis using the “Core Analysis” function (Fisher’s Exact Test (FET) *p* value: 1e−03). The upstream regulators were predicted by IPA using the default settings.

### ChIP-seq data analysis

ChIP-seq data sets, generated using PARIS WT in SH-SY5Y cells ‘in-house’, and the public (GSE120539)^24^ datasets, are composed of treatment and control samples, each with one replicate only. For each sample, after quality control as mentioned above, reads were separately aligned to the latest human genome (NCBI; GRCh38) using Bowtie with the parameter “-m 1” to retain uniquely mapped reads only. The R packages ChIPQC v1.20.0 and PhantomPeakQualTools v1.2.2 were used to confirm the quality of the aligned bam files based on the ChIP-seq guidelines by ENCODE consortium (NSC >  = 1.05; RSC >  = 0.8, Qtag = {− 2,− 1, 0,1,2})^25^. QC-confirmed bam files were subject to differential peak calling using MACS v1.4.2^26^ with the following parameters: -t Sample.sam -c Control.sam –format SAM -g hs -B -S– call-subpeaks. Peak annotation and visualization were done using the R packages ChIPseeker v1.20.0 and clusterProfiler v3.12.0, respectively. Tests for over-representation of gene ontology (GO) terms were performed using the R package ReactomePA v1.28.0 or the “goANA()” function in the R package limma v3.40.6. The gene list enrichment analysis platform, EnrichR v01.07.2020^27^, at https://amp.pharm.mssm.edu/Enrichr/ was used with the following libraries in this study: BioCarta MetabolicPA (2016) and Transcription Factor PPIs. STRING v11 protein association network analysis was performed with a minimal interaction score of 0.400 (FET *p* value: 1e−03)^28^. UCSC Genome Browser^29^ with custom tracks option enabled was used for visualization of peaks along the promoters and gene bodies. RSAT v2018^30^ and HOMER v4.11 (“findMotifs.pl” command)^31^ were used to perform binding motif analysis (BMA) with default parameters against the vertebrate and human backgrounds for motif enrichment, respectively. Yet, for in-house ChIP-seq dataset the min, the max, and the mean peak lengths were found to be excessively long for a regular BMA. Toward this end, the input peak sequences were restricted to include only summit of the peaks (i.e. + /− 100 bp.s).

### Code availability

The analysis pipelines and custom-made scripts presented in this manuscript are available from the corresponding author on reasonable request.

### Statistics

TRAP-seq dataset is composed of a total of 10 (TRAP control: 3, PARIS wild type: 3, PARIS mutant: 3, and whole brain: (1) replicate samples representing 4 different sample groups. A parametric *F*-test was used to analyze differential expression (significant changes in mean gene expression) by Ballgown while a Wald test or a likelihood ratio test (LRT) used by DESeq2 depending on the number of classes tested. A model-based analysis of MACS for peak calling is elaborated elsewhere^26^. As for the rest of analysis pipelines, 2-tailed, 2-sample Student’s *t *test was used to analyze the experimental and control groups in all in silico assays performed in this study. Fisher’s Exact Test or an alternate hypergeometric test was used for functional enrichment while a hypergeometric test (in HOMER) or a binomial or a chi-squared test (in RSAT) used for motif enrichment within the scope of this study. A *p* value or FDR of less than 0.05 was considered significant. Type of data distribution and validity of required assumptions were verified before statistical analysis in this work.

### Ethics declarations

Competing interests Statement: Patents related to this work include WO2017161155A1 “Methods for preventing or treating Parkinson’s disease by the farnesylation of PARIS.” T.M.D. and V.L.D are founders of Valted, LLC and hold an ownership equity interest in the company. These arrangements have been reviewed and approved by the Johns Hopkins University in accordance with its conflict of interest policies.

## Results

### Generation and characterization of hPARIS transgenic D. melanogaster

**Figure 1:**
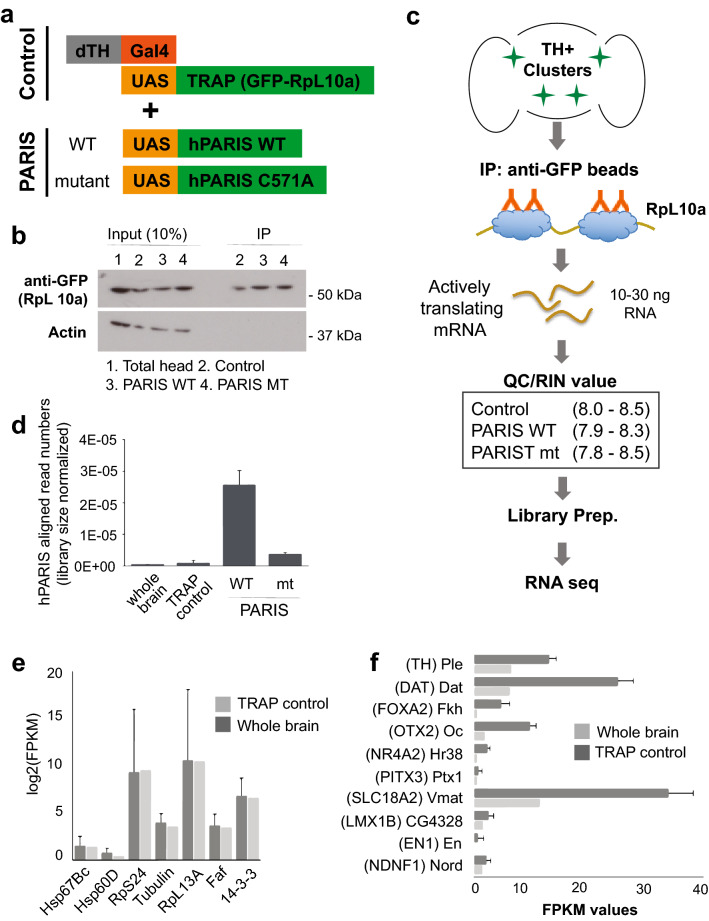
TRAP enables cell type–specific isolation of RNA from dopamine neurons. (**a**) The customized approach here for isolating RNA from dopamine (DA) neurons in Drosophila is based on use of human PARIS (hPARIS) expression construct to induce PD phenotype in fly and GFP-tagged ribosomal protein L10a (GFP-L10a) to specifically target actively translating mRNAs. (**b**) Immunoblotting results showing the expression of GFP-L10a fusion protein only for a qualitative assessment. The original membranes were cut prior to hybridization with antibodies during blotting. (**c**) TRAP-seq protocol. RNA integrity numbers (RINs) of around 8 or more demonstrating a high RNA integrity before sequencing. (**d**) Library size-normalized read counts calculated for hPARIS gene in fly genome, with PARIS wild type taking the lead as expected. (**e**) FPKM-normalized expression levels of a randomly selected group of fly housekeeping genes demonstrating TRAP protocol did not cause a global change in the transcriptome. (**f**) FPKM-normalized expression levels of DA neuron-specific biomarker genes significantly enriched in TRAP control. All RNA-seq based expression data presented here were expressed as mean ± standard error of the mean (SEM) values, for each condition studied.

To investigate the breadth and the extent of transcriptomic changes caused by human PARIS (hPARIS, ZNF746) gene in fly dopaminergic (DA) neurons, the double transgenic *UAS-GFP::RpL10A; UAS-PARIS-WT* and *UAS-GFP::RpL10A; UAS-PARIS C571A* lines were crossed with *dTH-GAL4* line to perform TRAP-seq (Fig. 1a, Supplementary Fig. 1). This technique allows for quantification of actively translating mRNAs by immunoprecipitating the ribosomes using a GFP-specific antibody in transgenic cells expressing the ribosomal protein L10a and GFP (GFP-L10a) fusion. A previously-established protocol for both generating and characterizing the transgenic fly lines, TRAP control, PARIS wild type (WT), and PARIS C571A mutant, used in this study^5^ was modified to enrich for TH( +) DA neurons expressing transgenic constructs (Fig. 1a, Supplementary Fig. 1). GFP-L10a briefly crossed with *dTH-Gal4* line showed tissue-specific expression pattern^5^, and further confirmed by immunoblot experiment (Fig. 1b). Thus, TRAP allowed us to isolate mRNA selectively from DA neurons expressing PARIS transgenic constructs. Sequencing libraries were prepared using high quality of RNA samples with RNA integrity numbers (RINs) of around 8 or higher, and subsequently sequenced on Illumina HiSeq platform (Fig. 1c, Supplementary Fig. 2–3). The number of reads mapping to human PARIS gene in fly genome was computed for all different samples separately, with PARIS WT taking the lead as expected (Fig. 1d). In a comparison between TRAP control and naïve whole brain samples, a randomly selected group of fly housekeeping genes demonstrated that TRAP protocol did not result in a global change in the transcriptome (Fig. 1e). Given the same comparison DA neuron-specific biomarker genes were found significantly enriched in TRAP control (Fig. 1f), confirming the quality of experiments with unique transcriptomic signatures from fly dopaminergic neurons.

### Two pairwise comparisons shed light on PARIS-driven global expression changes in fly DA neurons

**Figure 2:**
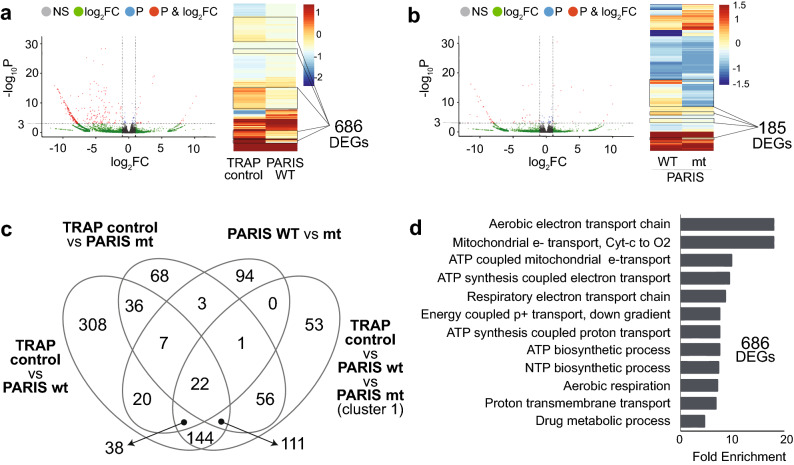
Two pairwise comparisons shed light on PARIS-driven global expression changes in fly DA neurons. (**a**-**b**) Only “TRAP control versus PARIS wild type” and “PARIS C571A mutant versus PARIS wild type” pairwise comparisons with a special focus on transcriptional suppression generated biologically meaningful outcome. (**c**) A Venn diagram^19^ showing only for these two comparisons mentioned here majority of the DEGs that are downregulated are comparison-specific, not shared as in the rest of the comparisons. (**d**) Functional enrichment results of 686 DEGs identified as downregulated from the “TRAP control versus PARIS wild type” comparison, implying mitochondrial dysfunction underlying PARIS phenotype.

In addition to transcriptome profiling of DA neurons in fly expressing hPARIS WT exogenously, we intended to investigate the regulatory role of the transcriptionally inactive mutant of PARIS, substituting cysteine for alanine at position 571 (C571A), published previously by our group^6^. This is because PARIS has, in addition to a pair of its DNA-binding domains, a KRAB domain that acts as a docking site for other DNA-binding proteins, implying an indirect means of transcriptional silencing by PARIS as an adaptor protein. Analysis of the differentially expressed genes (DEGs) identified from combinations of pairwise comparisons and 3-group comparison given the 3 different transgenic fly lines showed 686 DEGs in the TRAP control versus PARIS WT and 185 DEGs in the PARIS WT versus C571A mutant comparisons (Fig. 2a-b, Supplementary Fig. 4). A Venn diagram indicates the degree of overlap between the DEG lists identified in these comparisons (Fig. 2c). These 686 DEGs downregulated by PARIS WT were used for functional enrichment to confirm mitochondrial dysfunction in PARIS phenotype (Fig. 2d, Supplementary Fig. 5).

### Multifactorial transcriptomic changes induced by PARIS identify PPARγ as a master regulator

**Figure 3:**
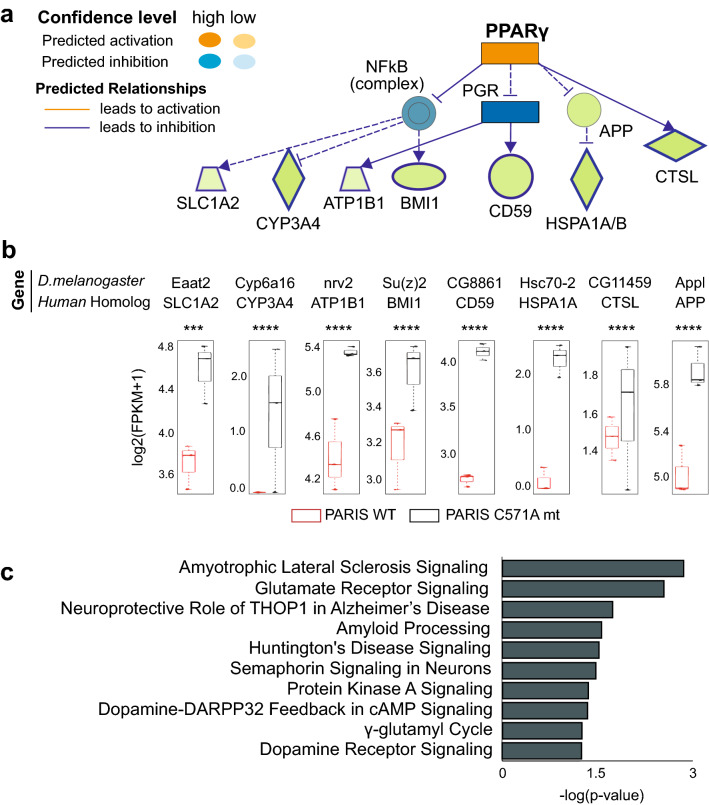
Multifactorial transcriptomic changes induced by PARIS identify PPARγ as a master regulator. (**a**) The PPARγ network enriched in the input dataset (i.e. human orthologs of the DEGs identified as downregulated from “PARIS C571A mutant versus PARIS wild type” comparison) using Causal Network Analysis (CNA) tool in IPA^34^. Blue arrows indicate a direct, suppressive relationship (such as causation, phosphorylation, and so on) while dashed arrows indicate an indirect relationship. Blue dashed lines without arrow indicate an indirect inhibition, likely by ubiquitination. Green nodes represent user-supplied genes (that is, downregulated in PARIS wild type) while the other nodes are predicted genes. Dark blue shading means the node is predicted to be inhibited by its regulator with high confidence (such as PGR) while pale blue shading means inhibitory prediction with low confidence (such as NFkB). Network shapes: rectangle (nuclear receptor), vertical diamond (enzyme), horizontal diamond (peptidase), ellipsis (transcription regulator), trapezoid (transporter), circle (other). Enrichment (FET) *p* value threshold: 1e−03. (**b**) The expression profile of the user-supplied genes in the PPARγ network in (**A**) with statistical significance values: ***p ≤ 0.001, ****p ≤ 0.0001. As expected, all these genes with plotted expression profiles are downregulated with high significance in PARIS wild type with respect to the mutant. (**c**) Enriched canonical pathways with a strong emphasis on neurodegeneration identified by Canonical Pathway Analysis (CPA) module in IPA^34^ taking the same human ortholog genes in (**A**) as input.

PARIS, which was identified as a novel substrate and a likely hub molecule in PD molecular network, downregulates peroxisome proliferator-activated receptor gamma (PPARγ) coactivator -1α (PGC-1α) to inhibit mitochondrial biogenesis^5,6^. Like the rest of the PPAR family TFs, PPARγ is also subject to: (1) transcriptional co-activation by PGC-1α, (2) heterodimerization with retinoid-X-receptor-α (RXRA) for DNA binding in a sequence specific manner on the promoter region of the target genes^13,32,33^.Thus, to explore functions of PARIS in nucleus as a trans-acting factor with extra domains for other DNA binding elements, we decided to apply Causal Network Analysis (CNA) tool on the IPA platform and investigated if PARIS pairs with other regulatory element(s) during gene regulation or if PARIS itself regulates the expression of other TFs in a cascade-like manner. Within the scope of this analysis upstream regulator is indicative of the TF activity explaining gene expression changes exhibited in input dataset, with the input being the human orthologs of the DEGs identified from “PARIS mutant versus PARIS WT” pairwise comparison. The IPA stands out as a tool based on prior knowledge of expected effects between transcriptional regulators and their target genes stored for this type of analysis. Yet, their knowledge base has been built exclusively for human, rat, and mouse queries so we performed strict one-to-one ortholog mapping for the fly DEGs in human, as suggested by the IPA guidelines^34^. PPARγ ranked first as the master regulator driving the observed expression changes caused by PARIS transcriptional activity (Table 1), supporting our previous findings that PARIS acts on PGC-1α along the NRF1/2-TFAM axis to intervene in mitochondrial biogenesis^6^. The PPARγ network enriched in the input list of DEGs is composed of 12 genes, 8 of which come from the input list (green nodes), of a depth of 2 with a 33% enrichment ratio, mostly of direct interactions (straight edges) (Fig. 3a). These 8 DEGs had a varying range of downregulation (FC: 1.9, > 10.0, 2.1, 1.4, 2.3, > 10.0, 1.3, and 1.9 respectively) with high statistical significance (*p* value up to 10^–24^) (Fig. 3b). As for the rest of the predicted master regulators listed in Table 1, the causal networks had full of indirect interactions at all regulatory layers predicted and contributing genes with relatively low significance (Supplementary Figs. 6–7). As a part of the IPA, Canonical Pathway Analysis (CPA) was also conducted to associate the same human ortholog genes with the known pathways in Ingenuity's Knowledge Base, which supports our previous finding that DNA binding capacity of PARIS is required to induce PARIS-driven neurodegeneration in DA neurons (Fig. 3c). The entire list of enriched pathways, which also highlights the specificity of TRAP-based dopaminergic neuron targeting at the pathway level, is given in Supplementary results (Supplementary Fig. 8). Finally, KEGG pathway and “Associated Disease” inferences from DAVID analysis with the human orthologs of the DEGs identified from “TRAP control versus PARIS WT” pairwise comparison taken as input highlighted PD and mitochondrial functioning as the top deregulated signaling pathways, and the neurological and the psychiatric disorders as the underlying disease networks (Table 2, Supplementary Figs. 9–10). The same human ortholog genes identified from this second comparison were used for another round of CPA, associating mitochondrial dysfunction and NRF2-mediated oxidative stress response with PARIS transcriptional repression (Supplementary Figs. 11–12). Taken altogether, PPARγ in DA neurons appears to be a potential master regulator of transcriptomic changes driven by PARIS, leading to mitochondrial dysfunction.

**Table 1:** Top 3 causal networks predicted by IPA^34^ explaining expression changes in the input dataset.

Master regulator	Participating regulators	Depth	Predicted activation	Activation z-score	*p* value of overlap	Network bias-corrected *p* value	Target molecules in dataset
PPARγ	APP, NFkB (complex), PGR, PPARG	2		0.707	5.84E−04	2.1 E−03	APP, ATP1B1, BMI1, CD59, CTSL, CYP3A4, HSPA1A/HSPA1B, SLC1A2
KIF5B-RET	CHUK, ERBB2, ESR1, FGFR1, IKBKB, …	3	Inhibited	− 2.324	6.19 E−04	5.3 E−03	APAF1, APP, ARHGAP1, ATP1B1, BMI1, CD59, CDC23, CSAD, CYP3A4, …
MUC1	ADAM17, AKT1, CASP8, CASP9, CDKN1A, …	3		− 1.528	7.07 E−04	7.1 E−03	APAF1, APP, ARHGAP1, ATP1B1, BMI1, CASP2, CD59, CDC23, CSAD, …

The network bias-corrected *p* value of overlap (calculated using Fisher’s Exact Test, *p* value < 0.05) defines possible significant overlaps between the input genes and the known targets regulated by an upstream regulator.

**Table 2:** Top 5 “Disease” and “pathway” terms enriched by DAVID Knowledge analysis^21^.

Analysis	Category	Term	Count	%	*p* value	Benjamini
Disease	GAD_DISEASE_CLASS	NEUROLOGICAL	65	20	2.7 E−02	3.9 E−01
Disease	GAD_DISEASE_CLASS	PSYCH	47	15	2.9 E−02	2.3 E−01
Pathway	KEGG_PATHWAY	Parkinson’s disease	15	5.4	1.5 E−07	3.0 E−05
Pathway	KEGG_PATHWAY	Metabolic pathways	45	16	6.5 E−07	6.4 E−05
Pathway	KEGG_PATHWAY	Huntington’s disease	15	5.4	6.0 E−06	3.9 E−04
Pathway	KEGG_PATHWAY	Carbon metabolism	10	3.6	1.5 E−04	7.5 E−03
Pathway	KEGG_PATHWAY	Citrate cycle (TCA cycle)	6	2.1	1.6 E−04	6.0 E−03

Disease and KEGG pathways^23^ with Fisher’s Exact Test based *p* value < 0.05 were selected.

### Characterization of genome-wide PARIS binding sites in SH-SY5Y neuroblastoma cells

**Figure 4:**
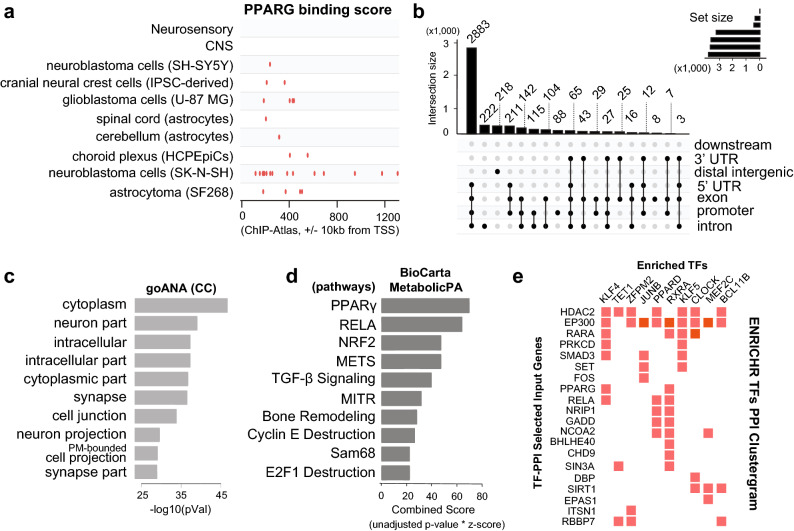
Characterization of genome-wide PARIS binding sites in SH-SY5Y neuroblastoma cells. (**a**) Human neuroblastoma cells provide a potential niche for PPARγ’s regulatory activities, justifying the choice of cell line for ChIP-seq experiment. (**b**) An upset plot^19^ showing the majority of significant PARIS ChIP-seq peaks overlap promoter region (− 3000, + 3000) of the genes in the human genome. (**c**) Enrichment of neuron-specific cellular components by PARIS taking the entire set of the peak-annotated genes as input. (**d**) Enriched metabolic pathways identified by BioCarta MetabolicPA module in EnrichR^27^ taking as input the significant peak-annotated genes that are also ranked by fold enrichment and that have a ChIP-seq peak overlapping the promoter region. Top 3 pathways include PPARγ and NRF2 pathways. (**e**) Enriched TFs identified by Transcription Factor PPI module in EnrichR^27^ taking the same genes in (**d**) as input. The most enriched TFs include PPARẟ and RXRA, the binding partner of PPARγ.

Next, we applied ChIP-seq as a means to build a genome-wide high-resolution map of the PARIS binding regions, and thereby, investigate transcriptional regulatory mechanism of PARIS in neuronal cells. The choice of human neuroblastoma cells to be used as a neurodegeneration model for this application stems from the knowledge that PPARγ appears highly active as a transcription factor in SH-SY5Y cell line (Fig. 4a). The majority of significant PARIS ChIP-seq peaks were mapped to promoter region (− 3000, + 3000) of the genes (Fig. 4b, Supplementary Fig. 13). Neuron-specific cellular components were enriched in the peak-annotated genes (Fig. 4c), confirming selective targeting of PARIS in neuroblastoma cells. Interestingly, the high number of peak-annotated genes identified at increased stringency (Table 3) implies how potent PARIS could be as TF in neuronal cells, unlike the low number of peak-annotated genes identified using the same set of parameters even at a lower stringency in HEK293 cells obtained from a public PARIS ChIP-seq experiment (Supplementary Fig. 14). To further explore the functional enrichment categories related to the most significant peak-annotated genes, a decision tree was made to rank, based on peak calling *p* value and then on fold enrichment, about 3400 genes to obtain a list of top 600 genes with a ChIP-seq peak overlapping exclusively the promoter region (Supplementary Fig. 15). As PPARγ is a key regulator in metabolic processes such as lipid and glucose metabolism, these shortlisted genes were used for BioCarta Metabolic Pathway Analysis on the EnrichR platform. The PPARγ pathway appears again as the most significant pathway enriched in the final list of peak-annotated genes while NRF2 Pathway ranked third (Fig. 4d, Supplementary Fig. 15). Taking the same list as the genes regulated by a hypothetical group of interacting TF(s), TF protein–protein interaction cluster-gram made on the same analysis platform revealed RXRA as the most enriched DNA binding element, followed by PPARẟ (Fig. 4e). RXRA is known as a well-defined PPAR family TF interacting partner^32,33^. Lastly, we used these top-ranked 600 peak-annotated genes for CNA and noted that Parkin is the top-most component of the regulatory network associated with the expression patterns observed in the input dataset (Supplementary Fig. 16). Overall, PPARγ-associated functional clusters, such as NRF-2-mediated oxidative stress, appear to form a crucial regulatory network in PARIS phenotype, supporting the observations from TRAP-seq analysis using fly model.

**Table 3:** Basic statistics describing in-house ChIP-seq outcome.

Parameters	Number of elements
*p* value	1E−25
FDR	0.05
Number of peaks detected	4244
Number of unique genes annotated	3738
Number of genes around promoter (TSS region = (− 3000, 3000))	3356

Peaks that could be assigned to the nearest genes (i.e. +/− 3 kb around TSS region) were used for downstream analysis. The estimated fragment size is 157 bp.s.

### PPARγ orchestrates PARIS-driven transcriptomic changes

**Figure 5:**
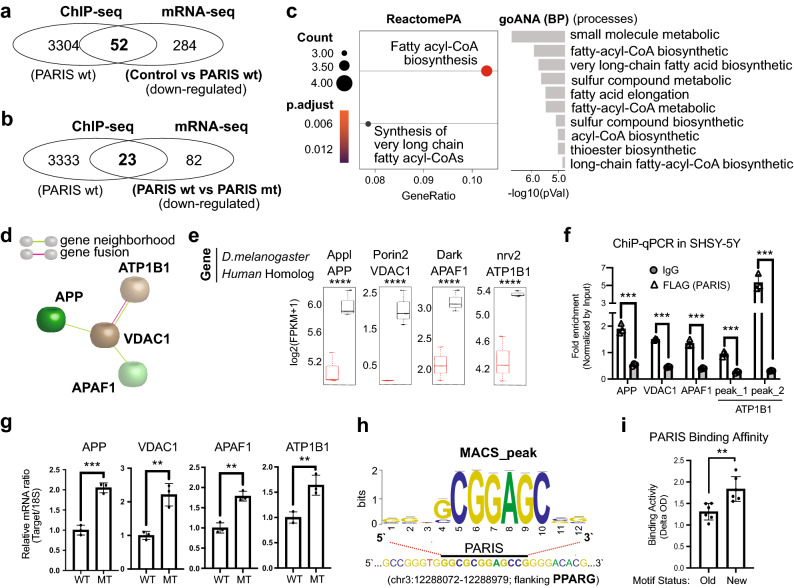
PPARγ orchestrates PARIS-driven transcriptomic changes. (**a**) A Venn diagram^19^ showing the shared genes between human orthologs of the DEGs identified as downregulated from “TRAP control versus PARIS wild type” comparison (TRAP-seq) and the entire set of the peak-annotated genes (ChIP-seq). (**b**) A Venn diagram^19^ showing the shared genes between human orthologs of the DEGs identified as downregulated from “PARIS C571A mutant versus PARIS wild type” comparison (TRAP-seq) and the entire set of the peak-annotated genes (ChIP-seq). (**c**) Ontology- and pathway-level enrichment results of the genes specified in (**a**). PPARγ-related GO terms are significantly enriched. (**d**-**e**) The STRING PPI network^28^ results of the shared genes specified in (**b**) and the expression profiles of the genes in this network. ***p ≤ 0.001, ****p ≤ 0.0001. The shared 23 DEGs successfully reproduced a part of the PPARγ network in Fig. 3a. FET *p* value threshold: 1e−03. Minimal Interaction Score: 0.400. Colored nodes: query genes (or proteins) and first shell of interactors. (**f**-**g**) ChIP-qPCR and RT-qPCR validation results of the target genes in (**d**). Both of the two distinct peaks associated with ATP1A1 gene were experimentally validated, together with the rest of the target genes. Statistical significances were analyzed with GraphPad Prism V7 software using Student’s unpaired t test. *P v*alues lower than 0.05 were considered significant. All qPCR-based expression data presented here were expressed as mean ± standard deviation values, for each condition studied. (**h**) PPARγ promoter region contains binding motif for PARIS. The k-mer significance = 123.19, the e-value = 6.5e−124, the number of genomic sites containing the motif = 4484. (**i**) The results of the colorimetric measurements that compare the binding affinities of PARIS to different motif sequences. PARIS has a significantly higher binding affinity to the new motif sequence identified within the scope of this study. **p ≤ 0.01.

In an attempt to combine two independent datasets to find common genes that are both physically regulated (from ChIP-seq) and significantly suppressed (from TRAP-seq) by PARIS, we identified 52 peak-annotated genes shared with the “TRAP control versus PARIS WT” comparison, and 23 genes with the “PARIS WT versus C571A mutant” comparison (Fig. 5a,b). Functional enrichment analysis revealed lipid metabolism associated terms such as fatty acid elongation and acyl-CoA biosynthesis as the key module explaining the functional trend for the first pairwise comparison above (Fig. 5c, Supplementary Fig. 17) while for the second the shared genes reproduced a part of the PPARγ network in Fig. 3a with significant differential expression (Fig. 5d,e). This smaller PPARγ network was then physically validated by ChIP-qPCR to confirm PARIS binding to these target genes (Fig. 5f) and by RT-qPCR to demonstrate PARIS-driven changes in their expression patterns (Fig. 5g). Because the rest of the 23 shared genes that were excluded from this network remained as disconnected nodes and could not be associated with functional terms related to PD, we set them aside for post hoc analysis. Apart from this, the motif analysis of PARIS-bound sequences demonstrated that the most frequently observed PARIS binding motif in this study, “GGCGCGGAGCCG”, was found at the promoter proximal site of PPARγ (Fig. 5h). Also, the core sequence of this motif was observed at the promoter site of NFE2L2 (NRF2) gene (Supplementary Fig. 18), implying a direct regulatory effect of PARIS on both genes. The previously identified PARIS binding motif by cyclic amplification and selection of targets (CAST) assay coupled with MACAW alignment^6^ was updated to a new, more refined motif. This refined motif was identified from two independent ChIP-seq datasets coupled with two technically different motif finder algorithms and is consistent with the original motif identified by CAST (Fig. S18A). Experimental validation of the newly identified consensus sequence and the old refined sequence are provided in Fig. 5i.

## Discussion

In this study, we aimed to elucidate the molecular mechanisms of how the accumulation of PARIS contributes to dopaminergic neuronal loss in human. We accomplished a part of this by mimicking PD phenotype in Drosophila model expressing human PARIS gene exclusively in dopaminergic neurons. The fact that PARIS zinc finger (ZNF) and KRAB domains, required for DNA and RNA binding as in many other TFs, are shared by approximately 800 other ZNF proteins in human necessitates the use of model organisms with no known similar domains to study these proteins. Similar issues need to be addressed for other mammalian systems, leaving fly as the choice of model organism that effectively reflects human brain complexity and disorders. Mirroring the diverse molecular composition and structure of human system, SH-SY5Y human neuroblastoma cell lines were used for the rest of this study. The rationale behind this switch from the fly model to a human cell line is as follows: (1) The primary finding of this study that PPARγ appears to define the mainstream of transcriptomic changes elicited by PARIS was made using human orthologs of fly genes, (2) Given this main finding, fly orthologs of human PPARγ, such as Hr96 and Eip75B, to be used in validation studies are so poorly annotated (with an identity match of 27% or less and prediction scores of 2/15 and 4/15, respectively^35^) that moving forward with these PPARγ orthologs would not be as informative, (3) Human neuroblastoma cells have been previously introduced as a potential niche for PPARγ’s regulatory activities (Fig. 4a)^36^, suggesting a good starting point to investigate possible changes in PPARγ-driven transcriptional regulation triggered by PARIS suppression.

The major finding of this work is the observation that PPARγ acts as a potential master regulator of transcriptomic changes induced by PARIS in fly DA neurons. This, in turn, is in line with our previous finding that PARIS acts primarily on PGC-1α to lead to neurodegeneration in PD phenotype^6,37^. Accompanying the regulatory role of PPARγ in PARIS molecular network is the deregulation of a set of genes involved in mitochondrial homeostasis. Here, we used a joint approach combining two complementary methods, TRAP-seq and ChIP-seq, to draw a regulatory landscape of PARIS target genes. In this sense, TRAP method was applied to isolate actively translating mRNAs selectively from DA neurons, with each sample successfully representing a distinct transcriptome profile (Supplementary Figs. 19–22). Though working with orthologous genes at the expense of ambiguity during cross-species predictions remains an unsolved issue, the identified functional clusters and molecular networks within the scope of this work appear to highlight PPARγ signature in PARIS molecular network at many distinct (e.g. genome, pathway, ontology, and regulatory network) levels when using these two approaches, both separately and combined.

Considering the expression profiles of the 3 transgenic fly lines in this study, we have tested all possible combinations of pairwise comparisons, as well as the 3-group comparison, within the scope of TRAP-seq data analysis. However, only two pairwise comparisons appeared to reflect the domain-specific effects of PARIS due probably to the statistical nature of our approach. For instance, we have experimentally shown in previous studies that PARIS C571A mutant rescues the phenotype induced by PARIS-driven transcriptomic changes^5,6,37^. In contrast to these in vitro and in vivo findings, our 3-group comparison results presented here do not seem to provide further evidence to corroborate this conclusion directly. Though “TRAP control versus PARIS WT” and “PARIS mutant versus PARIS WT” pairwise comparisons separately support this conclusion (in broad terms), as a caveat of our approach this previously observed expression pattern (i.e. transcriptionally suppressed in PARIS WT and restored back to normal levels in PARIS mutant fly lines) to describe the phenotypic changes in PD models could not form a significant cluster due to the presumably low number of DEGs with this expression profile identified in the 3-group comparison (Supplementary Fig. 23). The resulting clusters (Supplementary Fig. 24) could not be associated with functional terms related to PD, either. Similarly, we have experimentally reported in both fly and human SH-SY5Y cells significant downregulation of PGC-1α, and PGC-1α-responsive genes by PARIS within the scope of the same previous studies^5,6^. Though we did see a trend toward downregulation by the wild type and rescue by the mutant version of PARIS for PGC-1α in this work, this trend failed to reach statistical significance (*p* value: 0.178). These phenomena can typically be considered as false negative results attributable to the small sample size and the statistics used. In technical terms, the lack of statistical power does not necessarily mean such expression patterns do not exist in the input dataset at all. After finding that both gene-level and transcript-level differential expression analyses yield similar outcomes in this respect, we decided to continue with the only comparisons that are both statistically and biologically informative. Interestingly, the majority of the DEGs identified in any of these comparisons tend to be downregulated (Fig. 2a-b, Supplementary Figs. 4 and 23), confirming the suppressive nature of PARIS at large. As described above, only for these two pairwise comparisons central to our study majority of the DEGs that are downregulated are comparison-specific, not shared as in the rest of the comparisons (Fig. 2c), implying a unique molecular mechanism underlying the phenotype induced by PARIS accumulation in PD. Lastly, identifying PPARγ as the key regulatory element of PARIS-led transcriptional repression particularly in “PARIS C571A mutant versus PARIS wild type” comparison highlights how critical it is to preserve DNA binding capacity of PARIS for the proper functionality. This might rule out the possibility that PARIS repression of PPARγ is independent of PARIS-DNA interaction through the blockage of PPARγ nuclear transport or some similar cellular processes, as stated before^6,38^.

One perplexing observation is that being the root node of the network, PPARγ appeared activated (orange central node in Fig. 3a), which was expected to be downregulated by PARIS. One possible explanation is that the absolute “Activation Z-score” used to infer the activation state of a putative regulator (i.e. whether the regulator is activated or inhibited)^39^ was less than “2” (that is, |z|= 0.707) for PPARγ, meaning the prediction of PPARγ activation state is not statistically significant. Indeed, activation Z-score is not recommended to use if the putative regulator is flagged “biased” by the analysis tool, which is valid for PPARγ. That is why the state of “Predicted Activation” column in Table 1 for PPARγ appeared undetermined, neither activated nor inhibited unlike some of the other top-most master regulators predicted from the same input data. As a result, to rule out the possibility of a false positive outcome we decided to move ahead with further analysis, ignoring the activation status predictions and focusing solely on the network enrichment independent of the edge weight or sign, excluding regulation direction for the entire PPARγ network.

As can be seen in Supplementary Fig. 7, the fly ortholog of human PPARγ, such as Hr96, does not appear downregulated by PARIS. Apart from the poor annotation of human PPARγ in fly, all the evidence on PARIS-driven PPARγ deregulation collected so far can be explained using an alternate hypothesis. KRAB-domain of DNA-binding proteins like PARIS interacts with a scaffold protein, KAP-1, to recruit other regulatory proteins, assembling the KRAB-ZFP/KAP1 repressor complex and leading to heterochromatin formation for the transcriptional silencing of target genes^40,41^. Histone deacetylases (HDACs) are among the core components of this large protein complex. Considering that transcriptional activity of PPARγ is physically blocked inside the nucleus by the inhibitory interaction between HDAC4 and RXRA/PPARγ heterodimers in cortical neurons under oxidative stress^38^, PARIS in DNA-bound state as a part of the repressor complex might be suppressing PPARγ in a one-to-one interaction by physically preventing PPARγ from binding to DNA. Further research is underway to uncover these mechanistic aspects of the active competition for binding site occupancy between PARIS and PPARγ.

Collectively, a set of clues that suggests the promoter location of PPARγ in human genome can be seen in Supplementary Fig. 18. As can be seen in the same figure, PARIS appears to physically interact with the promoter region for a direct regulation of PPARγ expression as well, providing further insight into the involvement of PPARγ in PARIS molecular network. A key finding came from motif analysis of PARIS-bound sequences; we observed a PARIS binding motif at the promoter proximal site of PPARγ and NRF2, implying a direct regulatory effect of PARIS on the expression of both genes (Supplementary Fig. 18). Originally, the pattern discovery was intended for lower eukaryotes and bacteria since the dispersion of regulatory elements in more complex organisms over very large distances and the heterogeneity of their promoter compositions make pattern discovery inconvenient for higher eukaryotes. Yet, apart from confirming our findings from TRAP-seq analysis, obtaining very similar results from different datasets coupled with different predictive tools suggests achieving consistency for pattern discovery even in higher eukaryotes is possible. Also, experimental validation of this newly identified and more specific (12 nts) motif sequence to which PARIS has even higher binding affinity in comparison to the previously identified (7 nts) consensus sequence added another layer of evidence to the view that motif discovery is applicable to all species irrespective of the genomic complexities.

Lastly, the four common genes in Fig. 5d, which are both physically regulated (from ChIP-seq) and significantly suppressed (from TRAP-seq) by PARIS, were the subject of experimental validations in the final stage of this study. What makes these genes valuable for neurodegenerative and neuroprotective research is that they are mostly brain-specific genes that play critical roles in the maintenance of neuronal health. Amyloid Beta Precursor Protein, or simply APP, for example is a well-studied gene that is known to form the protein basis of the amyloid plaques found in the brains of patients with Alzheimer disease^42^. ATP1B1, on the other hand, encodes a key Na^+^/K^+^-ATPase that establishes and maintains the osmoregulation across the plasma membrane for proper neuronal excitability^43^. VDAC1-encoded protein is a central component of the outer mitochondrial membrane that functions as a voltage-dependent anion channel involved in transmembrane electron transport^44^. Apoptotic Peptidase Activating Factor 1, or APAF1, encodes a cytoplasmic protein that acts as a major player in the initiation of apoptotic protease cascade in a cytochrome c dependent manner^45^. As a whole, the critical tasks achieved by these PARIS target genes imply how critical it is to preserve a proper level of PARIS expression in DA neurons.

## Conclusion

The current study presents further evidence that PARIS acts along the PPARγ-NRF1/2-TFAM axis to compromise mitochondrial homeostasis and to induce neurotoxicity. In particular, PPARγ appears as the top-most component of the regulatory network governing PARIS-induced transcriptomic changes in neuronal cells. Nevertheless, whether PARIS acts on PPARγ, as well as NRF2, directly as suggested in this study or through PGC-1α or both requires further investigation. The *Drosophila* model with cell-type specific induction of human PARIS gene used in this work to mimic neurological disorders would continue to serve as a paradigm for future studies to unravel mechanistic underpinnings of the biology of PARIS, as well as other 800 human KRAB-zinc finger proteins.

## Supplementary Information

Supplementary Information 1.

Supplementary Information 2.

## Data Availability

Based on the MINSEQE standards^46^ TRAP-seq and ChIP-seq datasets have been deposited in the NCBI’s Gene Expression Omnibus (GEO) database under the accession GSE175556. Original membranes for Fig. 1b were cut prior to hybridization with antibodies during blotting. To provide specific detection of the target antigen, the full-size immunoblotting results are presented using input samples in Supplementary Fig. 25. All the gene lists discussed throughout the manuscript are also available as Supplementary Information File.
