# Supplementary material for: Integrative genome-wide analysis of dopaminergic neuron-specific PARIS expression in Drosophila dissects recognition of multiple PPAR-γ associated gene regulation
Source: Sci Rep. 2021 Nov 2;11:21500. doi: 10.1038/s41598-021-00858-7 (PMC8563805; doi:10.1038/s41598-021-00858-7)
Supplement: Supplementary file 1 — Supplementary Information 1. [file 41598_2021_858_MOESM1_ESM.pdf]

Integrative genome-wide analysis of dopaminergic neuron-specific PARIS expression in *Drosophila* dissects recognition of multiple PPAR-γ associated gene regulation.

Volkan Yazar[1,6], Sung-Ung Kang[1,2,6], Shinwon Ha[1], Valina L. Dawson[1,2,3,4*] & Ted M. Dawson[1,2,4,5*]

Corresponding Authors:

Ted M. Dawson, M.D., Ph.D.
Neuroregeneration and Stem Cell Programs
Institute for Cell Engineering
Johns Hopkins University School of Medicine
733 North Broadway, Baltimore, MD 21205, USA.
E-mail: tdawson@jhmi.edu

Valina L. Dawson, Ph.D.
Neuroregeneration and Stem Cell Programs, Institute for Cell Engineering
Johns Hopkins University School of Medicine
733 North Broadway, Suite 711
Baltimore, MD 21205
Email: vdawson@jhmi.edu

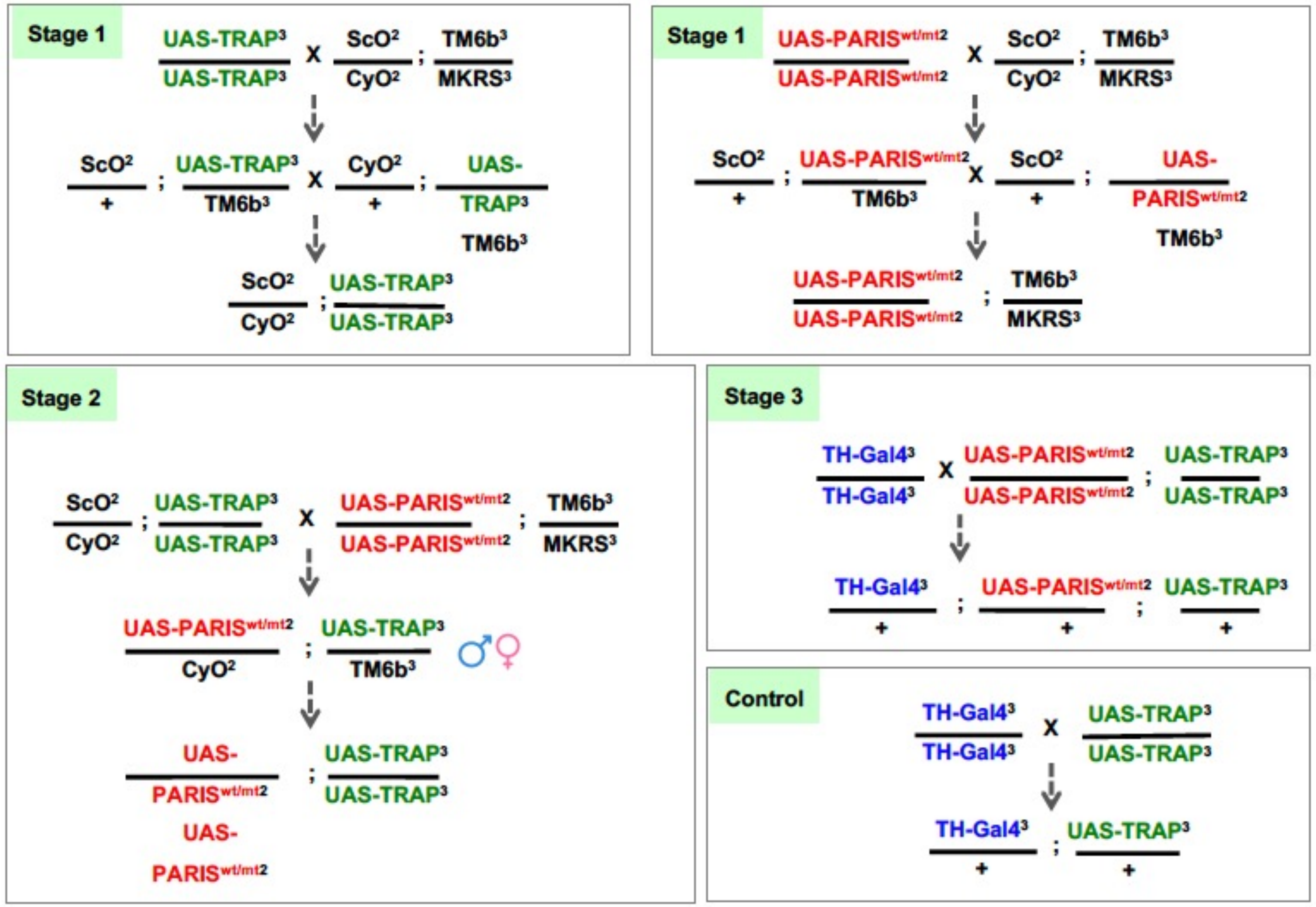

Supplementary Figure S1. Drosophila genetic line for TH-TRAP/TH-TRAP-PARIS WT and mutant (C571A). Illustration for the generation of *Drosophila* genetic lines for TH-TRAP/TH-TRAP-PARIS WT and mutatnt (C571A).

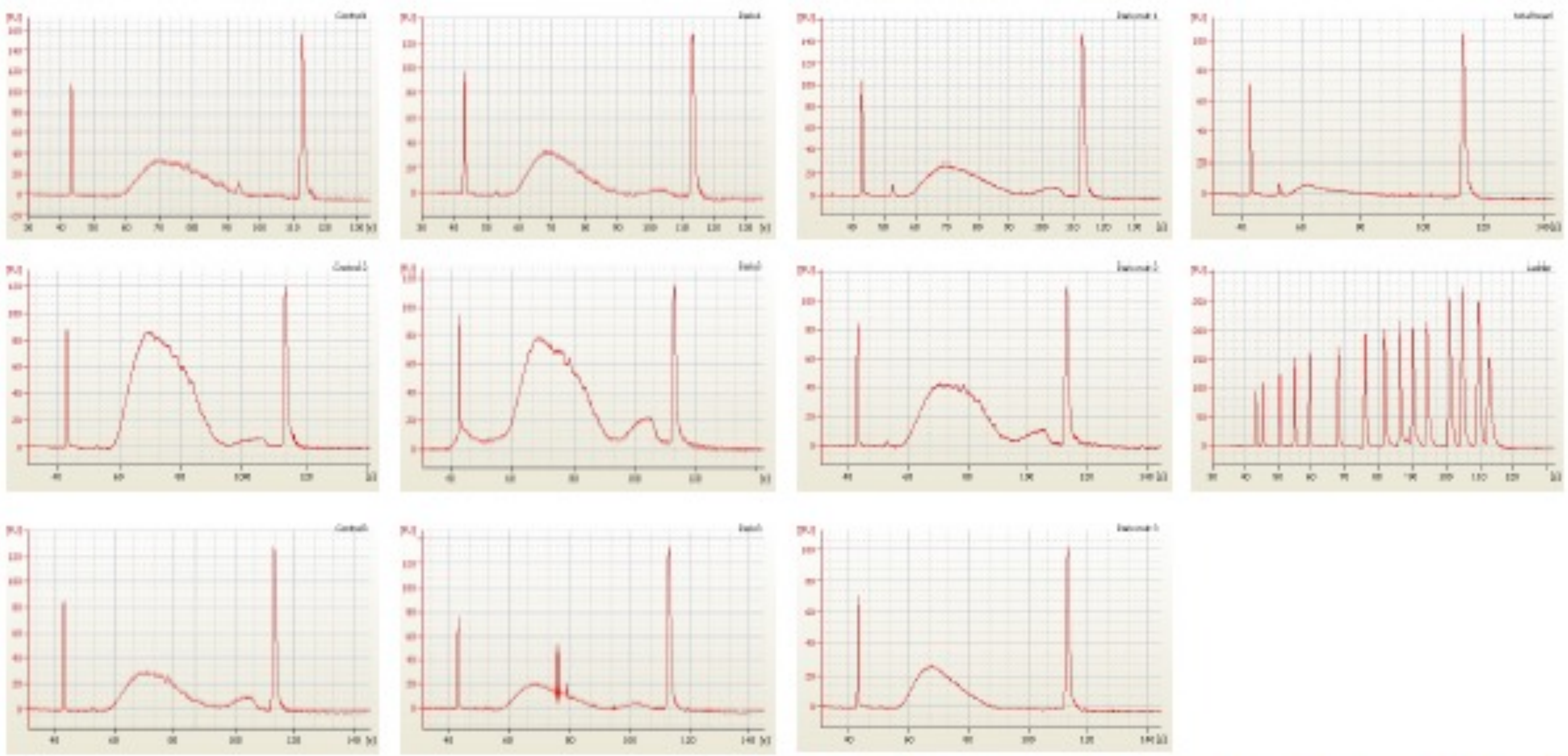

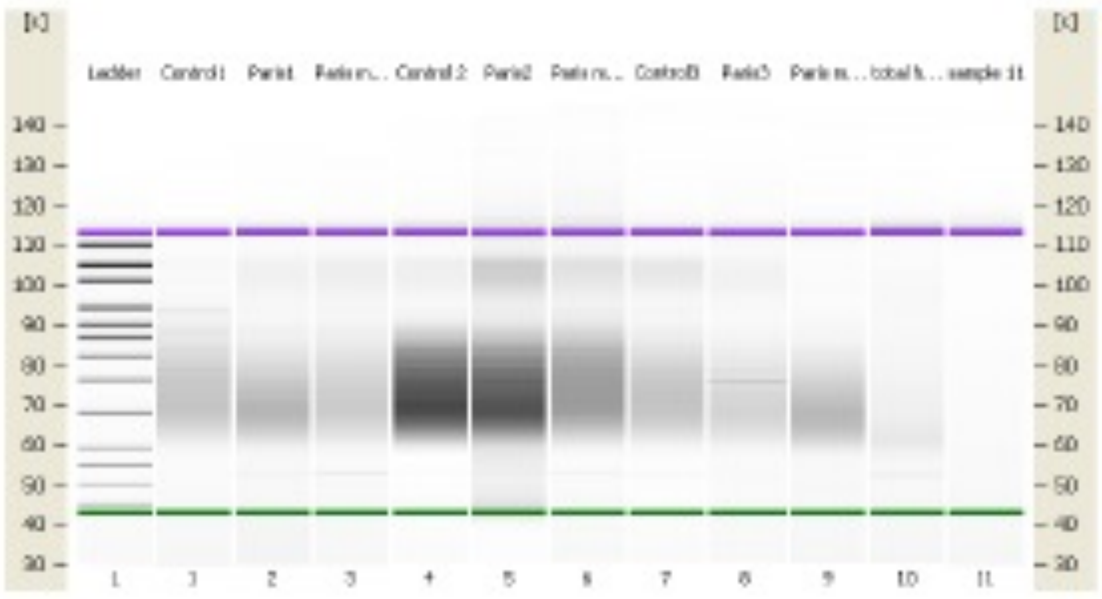

Sample #	Sample Name	Index (Sequence)	Concentration (ng/uL)	Average Fragment size (bp)
01	Control_S01	ATCACG	2.958	410
02	PARIS WT_S01	CGATGT	2.899	365
03	PARIS MT_S01	TTAGGC	2.378	392
04	Control_S02	TGACCA	8.515	387
05	PARIS WT_S02	ACAGTG	7.633	389
06	PARIS MT_S02	GCCAAT	4.848	411
07	Control_S03	CAGATC	3.247	390
08	PARIS WT_S03	ACTTGA	1.693	365
09	PARIS MT_S03	GATCAG	2.800	339
10	PARIS Whole Brain	TAGCTT	0.557	350

*Concentrations were measured using Agilent 2000 Bioanalyzer.
** Multiplex Oligos (index primers) from NEBNext Multiplex Oligos for Illumina).

Supplementary Figure S2. Quality of sequencing library. The quality control includes checking for sample concentration, sizing, purity, molarity (quantification), and integrity at multiple checkpoints, including the starting material, intermediate products, and final libraries.

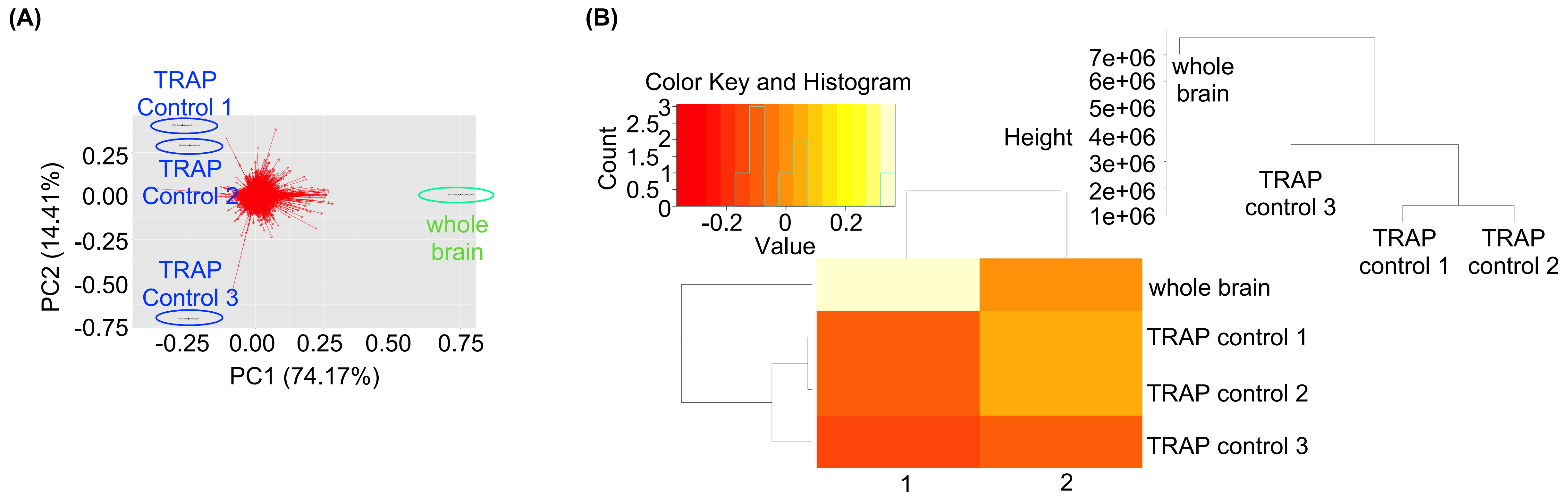

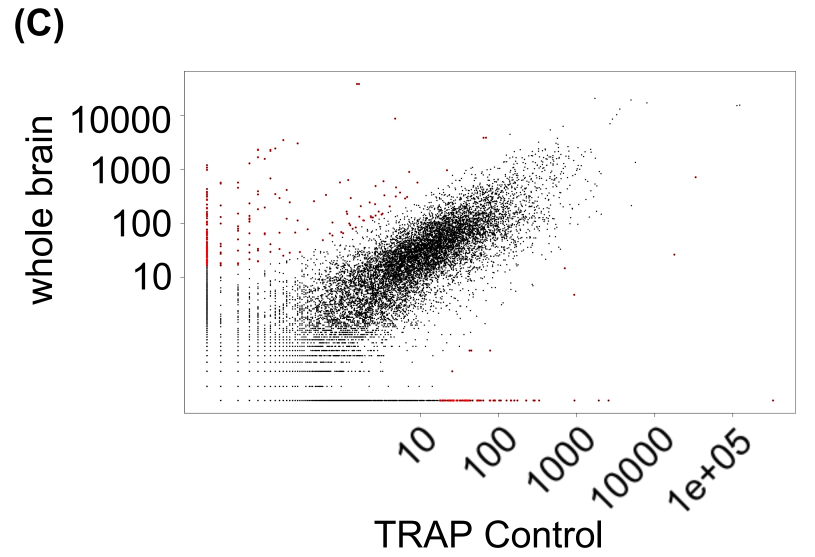

(D)

Analysis Tools		NOISeq-sim v2.28.0
Input data format		Raw Count
Input data features		Control:3x Replicate, Whole Brain:1x Replicate
q value	0.90	281
	0.95	165
	0.99	67
	0.999	20

Supplementary Figure S3. Exploratory plots depicting changes in expression levels between TRAP control and whole brain. Diagnostic plots illustrating the effect of TRAP protocol on fly transcriptome. (A) A PCA plot confirming the absence of systematic, unwanted transcriptomic changes between whole brain and TRAP control samples. (B) A correlation heatmap and hierarchical clustering results reveal at the first dimension that the replicates from TRAP control sample form a separate cluster. (C) A scatter gene expression plot showing overall distribution of the fly transcriptome with respect to TRAP control. (D) Differential expression analysis results of TRAP control vs. whole brain pairwise comparison. It is clear by the few number of DEGs identified at a range of NOISeq-sim-specific “q-value” thresholds that TRAP protocol does not have a global impact on gene expression in Drosophila. NOISeq-sim has been used particularly in this comparison because the whole brain sample has only one replicate to analyze.

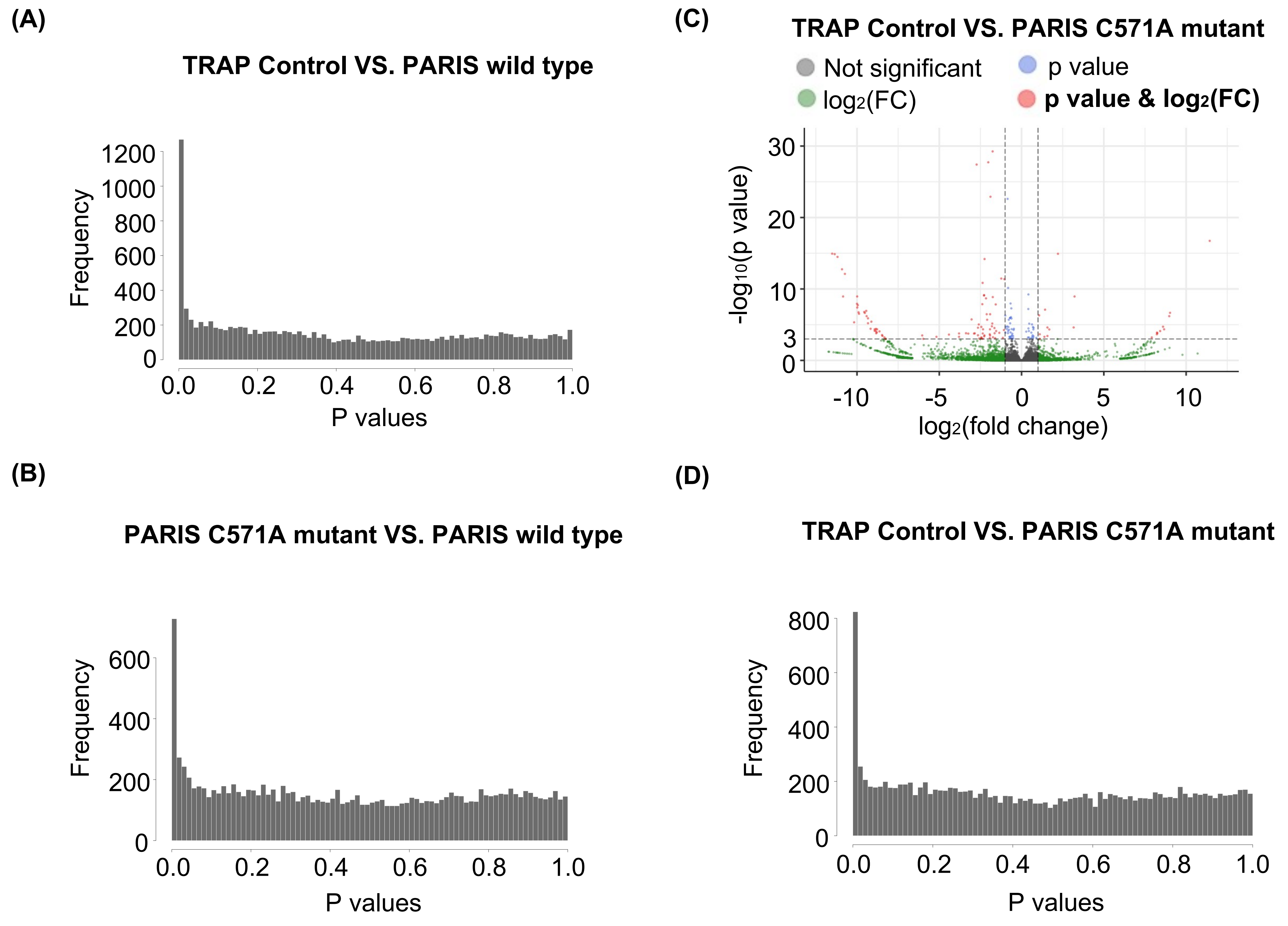

Supplementary Figure S4. Exploratory plots depicting changes in expression levels from TRAP saamples. (A, B, D) P-value histograms confirming the expected uniform distribution of null p-values. (C) A volcano plot showing significant genes with expression fold changes above the threshold.

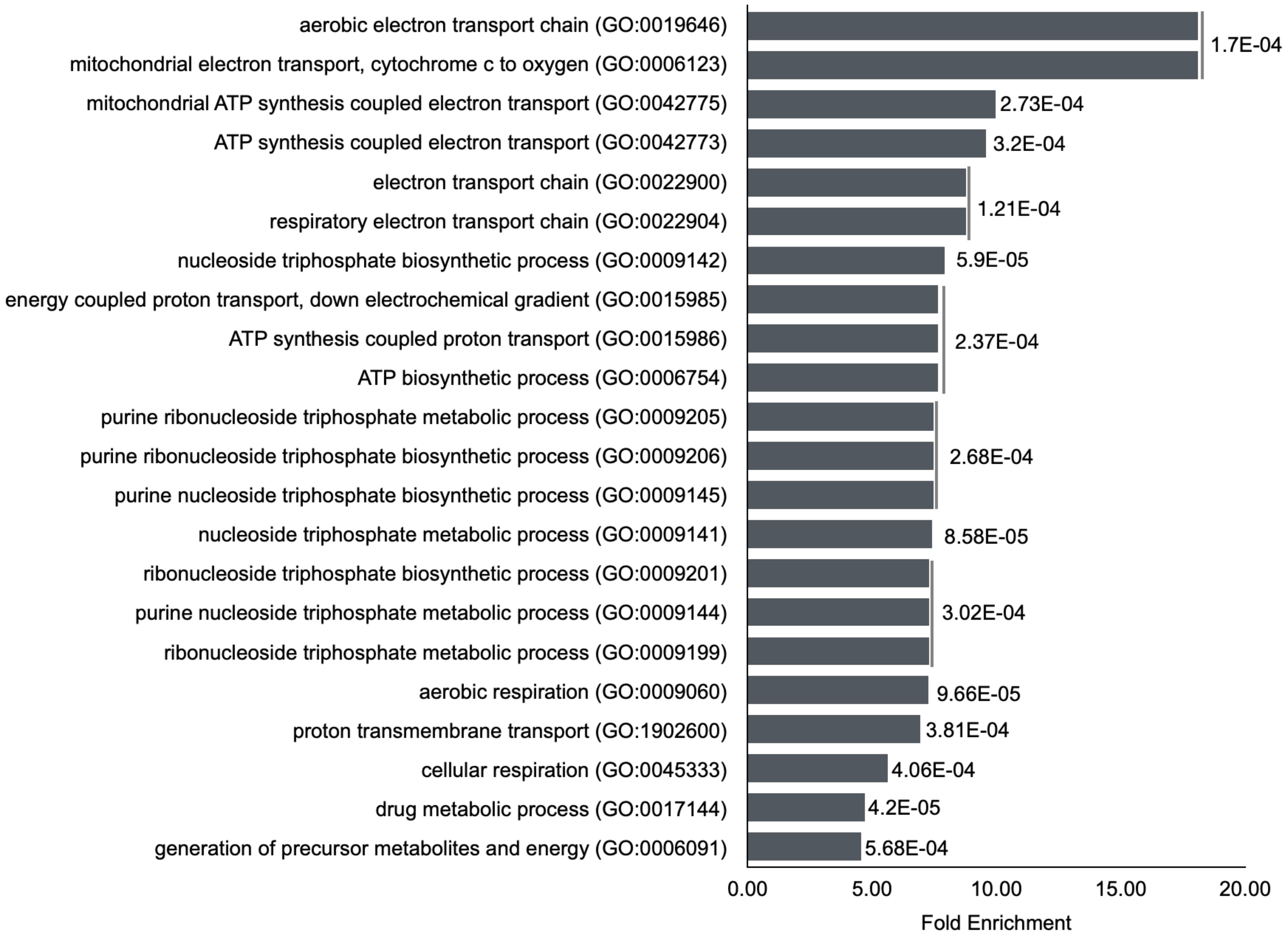

Supplementary Figure S5. List of functional enrichment results of the downregulated genes identified from TRAP control vs. PARIS wild type pairwise comparison.

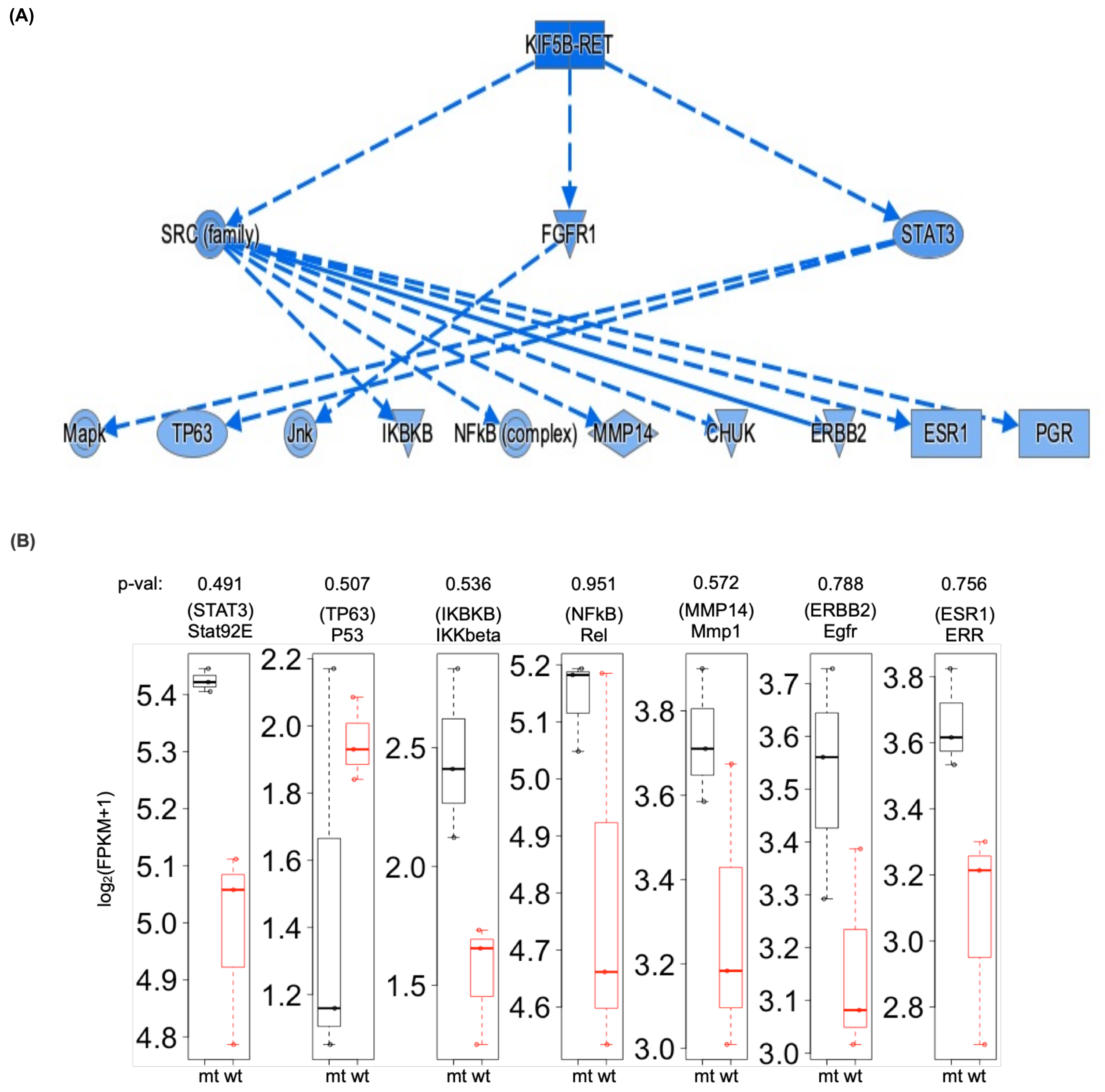

*The rest of genes in each network separately were filtered out during pre-processing due to insufficient read count

Supplementary Figure S6. KIF5B-RET networks enriched after CNA. Other master regulators than PPARγ predicted using causal network analysis by IPA have networks with full of indirect interactions and with components that have no significant expression changes. All nodes in (A) were quantitatively evaluated in (B).

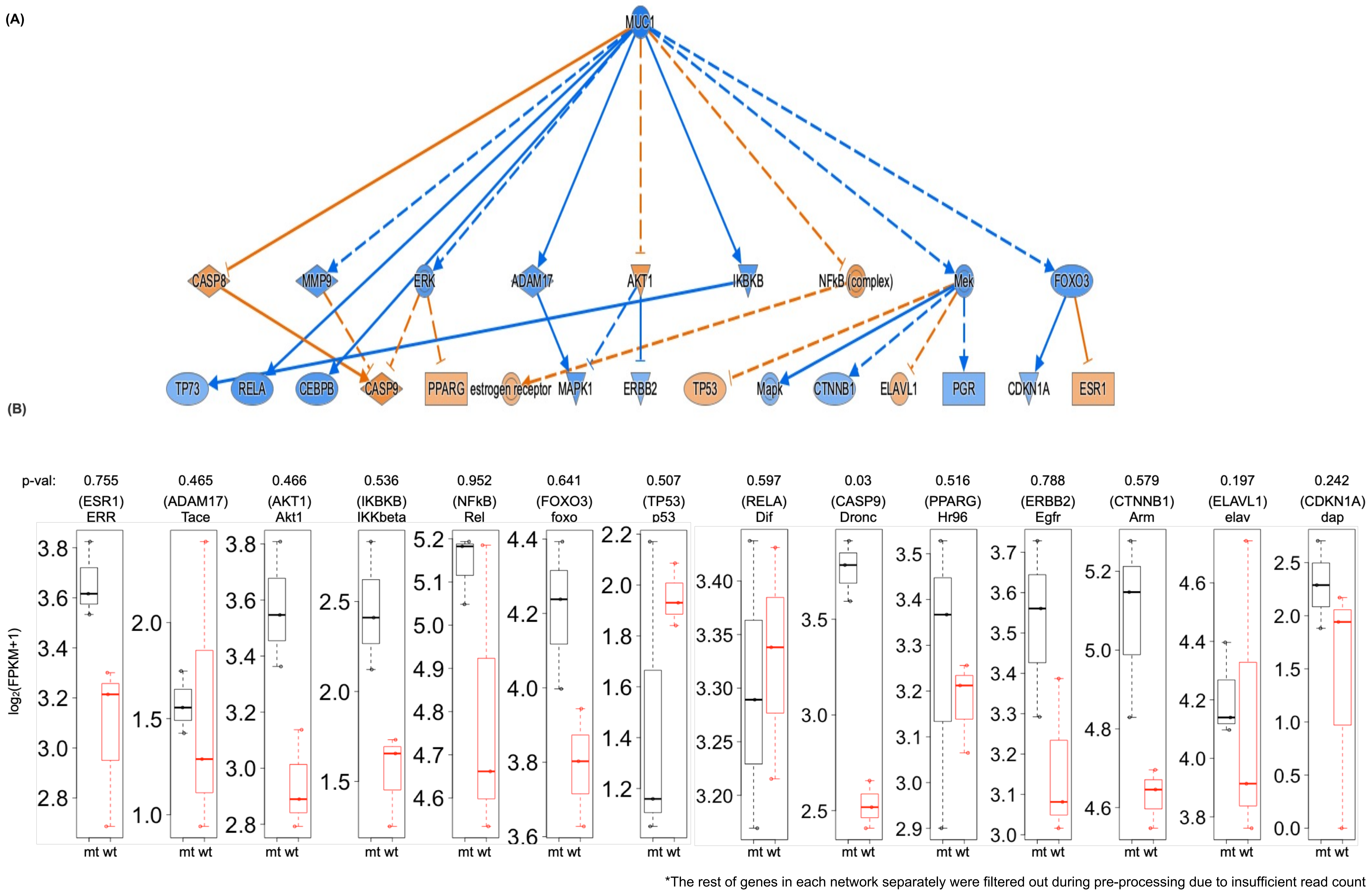

Supplementary Figure S7. MUC1 networks enriched after CNA. Other master regulators than PPARγ predicted using causal network analysis by IPA have networks with full of indirect interactions and with components that have no significant expression changes. All nodes in (A) were quantitatively evaluated in (B).

PARIS C571A mutant VS. PARIS wild type (down-regulated)

Ingenuity Canonical Pathways	-log(p-value)	Molecules
**Amyotrophic Lateral Sclerosis Signaling**	2.85E+00	APAF1,GRIK1,GRINA,SLC1A2
**Glutamate Receptor Signaling**	2.55E+00	GRIK1,GRINA,SLC1A2
**Methionine Degradation I (to Homocysteine)**	2.35E+00	MAT2A,PRMT1
**Sorbitol Degradation I**	2.3E+00	SORD
**Cysteine Biosynthesis III (mammalia)**	2.27E+00	MAT2A,PRMT1
**Taurine Biosynthesis**	2E+00	CSAD
**Protein Ubiquitination Pathway**	1.94E+00	CDC23,DNAJC30,HSPA1A/HSPA1B,HSPD1,UBE2E2
**Superpathway of Methionine Degradation**	1.87E+00	MAT2A,PRMT1
**S-adenosyl-L-methionine Biosynthesis**	1.82E+00	MAT2A
**Docosahexaenoic Acid (DHA) Signaling**	1.81E+00	APAF1,APP
**Neuroprotective Role of THOP1 in Alzheimer's Disease**	1.74E+00	APP,PRSS36,TPSAB1/TPSB2
**Role of Oct4 in Mammalian Embryonic Stem Cell Pluripotency**	1.67E+00	BMI1,PPP1R8
**TNFR1 Signaling**	1.61E+00	APAF1,CASP2
**Rapoport-Luebering Glycolytic Shunt**	1.6E+00	PGAM2
**Myc Mediated Apoptosis Signaling**	1.58E+00	APAF1,CASP2
**Amyloid Processing**	1.58E+00	APP,CSNK1A1
**Huntington's Disease Signaling**	1.52E+00	APAF1,CASP2,HSPA1A/HSPA1B,POLR2C
**Semaphorin Signaling in Neurons**	1.48E+00	ARHGAP1,RAC2
**Cardiac β-adrenergic Signaling**	1.47E+00	AKAP1,PDE1A,PPP1R14B
**Aldosterone Signaling in Epithelial Cells**	1.37E+00	DNAJC30,HSPA1A/HSPA1B,HSPD1
**Protein Kinase A Signaling**	1.35E+00	AKAP1,CDC23,MYL7,PDE1A,PPP1R14B
**Dopamine-DARPP32 Feedback in cAMP Signaling**	1.34E+00	CSNK1A1,GRINA,PPP1R14B
**γ-glutamyl Cycle**	1.27E+00	ANPEP
**Dopamine Receptor Signaling**	1.25E+00	PPP1R14B,SLC18A3

Supplementary Figure S8. List of IPA canonical pathway. The human orthologs representing downregulated fly genes identified from PARIS C571A mutant vs. PARIS wild type pairwise comparison. Neurodegeneration in dopaminergic neurons highlights the pathway analysis results.

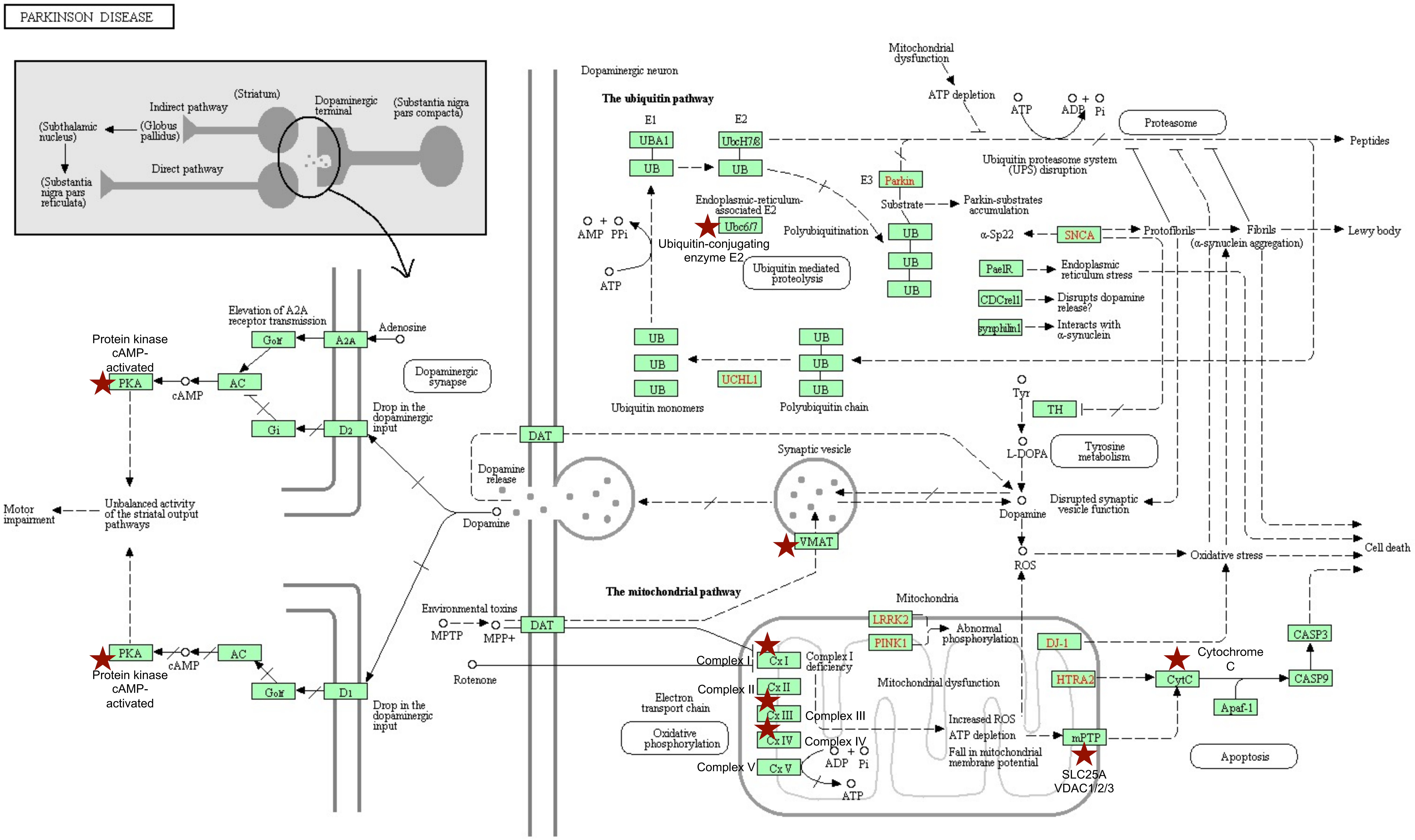

Supplementary Figure S9. Global view of the Parkinson's disease network with deregulated elements highlighted. KEGG pathway[23] and "Associated Disease" inferences from DAVID analysis with the human orthologs of the DEGs identified from "TRAP control vs. PARIS wt" pairwise comparison highlights PD and mitochondrial functioning as the top deregulated signaling pathways.

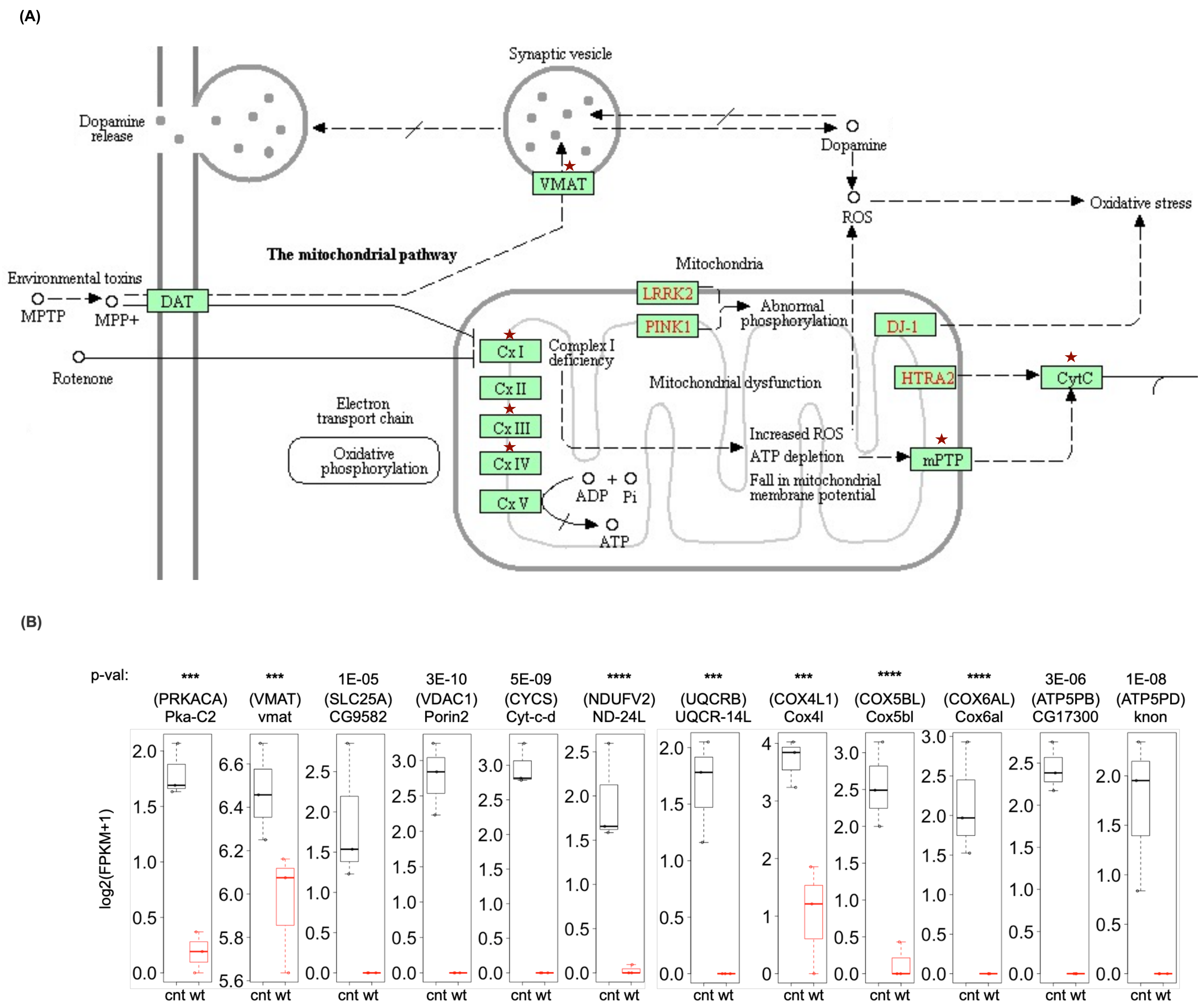

Supplementary Figure S10. Parkinson's disease is a molecular network regulated by PARIS. (A) The part of the KEGG[23] Parkinson's disease molecular network transcriptionally regulated by PARIS. (B) Nodes with a red star in Supplementary Figure S9 were quantitatively evaluated.

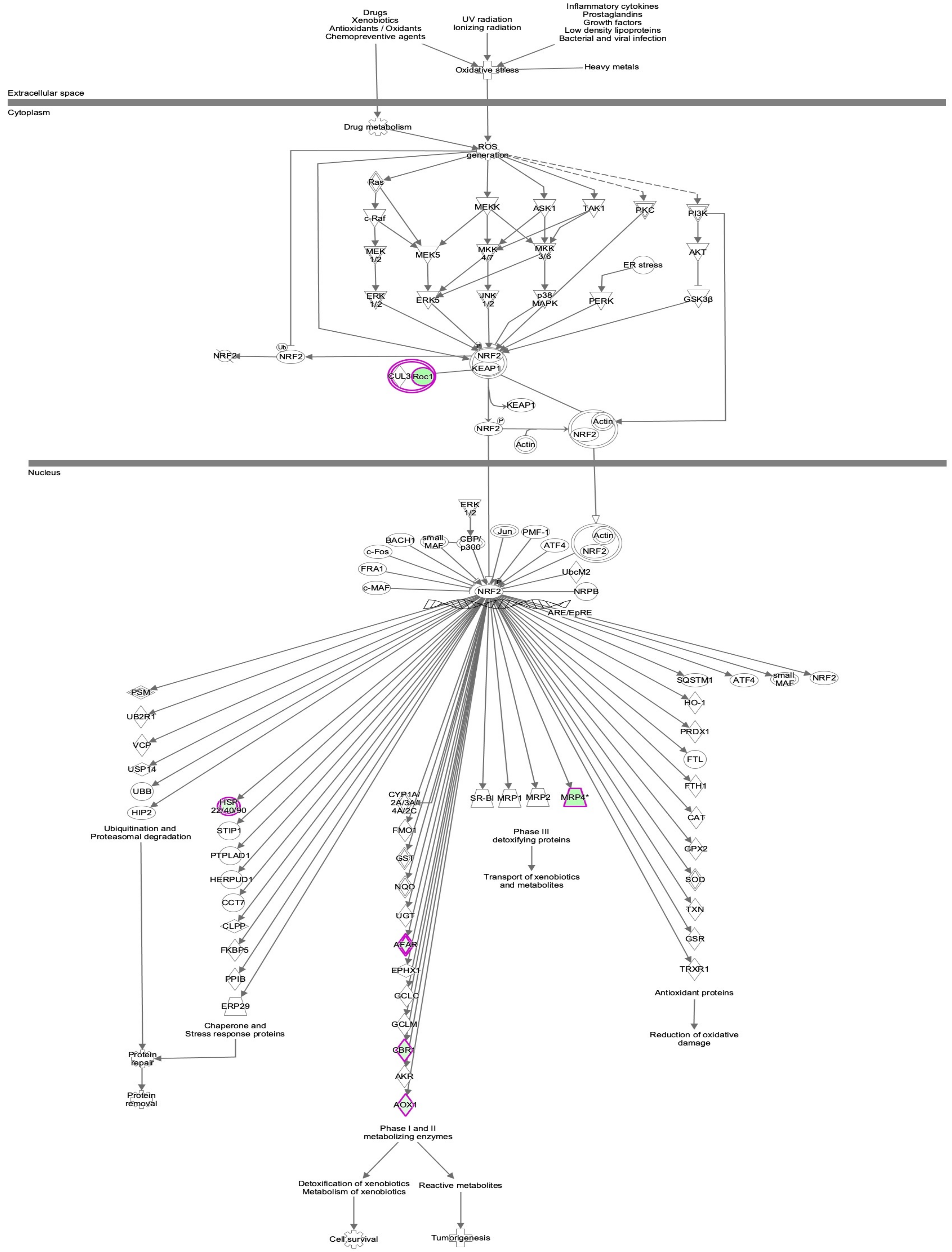

Supplementary Figure S11. Global view of NRF2-mediated oxidative stress response pathway with deregulated elements highlighted. IPA Canonical Pathway Results of the human orthologs representing downregulated fly genes identified from “TRAP control vs. PARIS WT” pairwise comparison identifies NRF2 pathway as a significantly deregulated pathway.

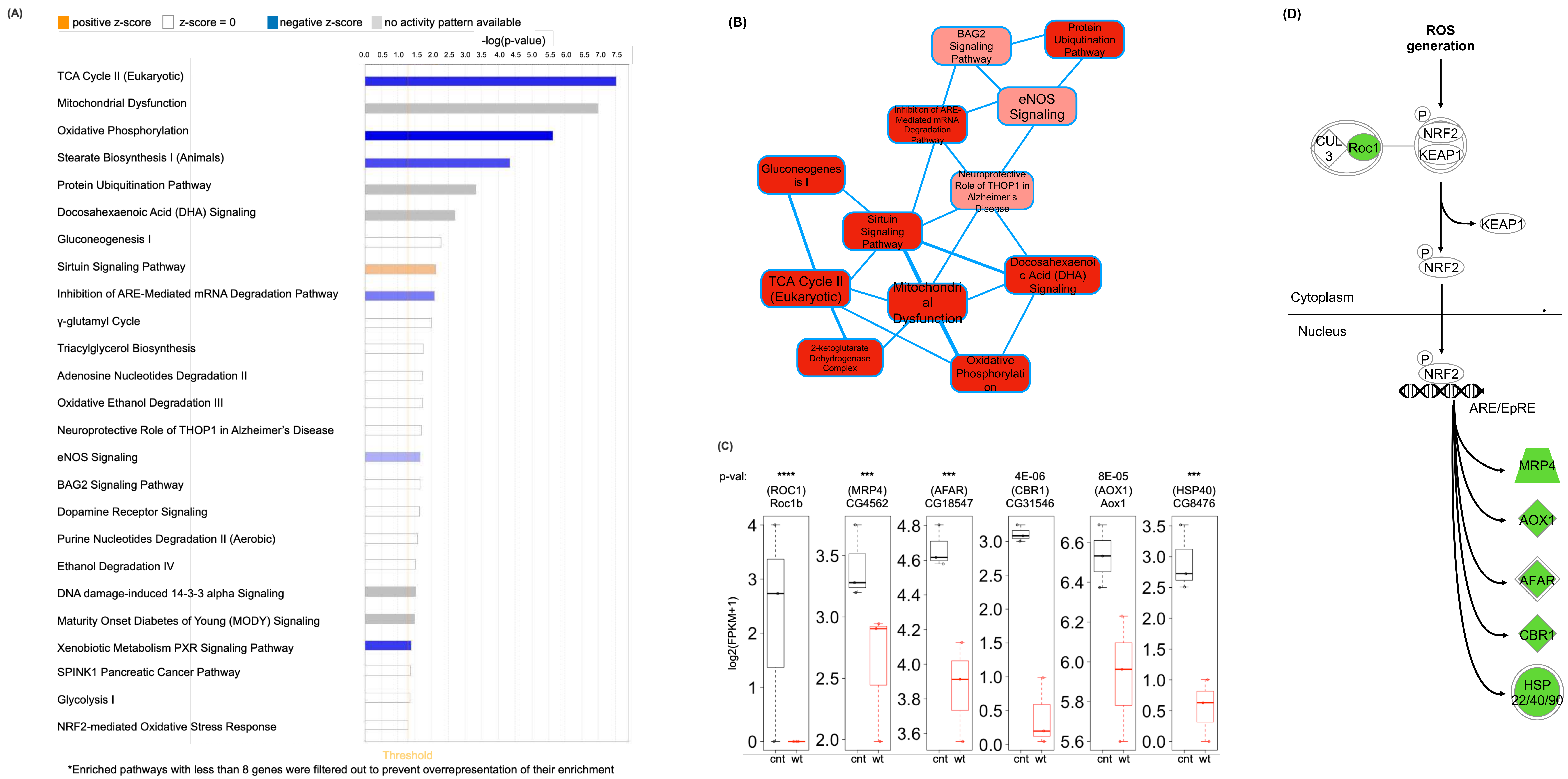

Supplementary Figure S12. NRF2-mediated oxidative stress response, a mitoprotective downstream signaling of PPAR-γ pathway, is regulated by PARIS. (A, B) IPA Canonical Pathway Analysis relates mitochondrial dysfunction and NRF2-mediated oxidative stress response with PARIS transcriptional repression. (C, D) Significant downregulation of NRF2-mediated Oxidative Stress Response Pathway elements are illustrated graphically. Green nodes in (D) were quantitatively evaluated in (C).

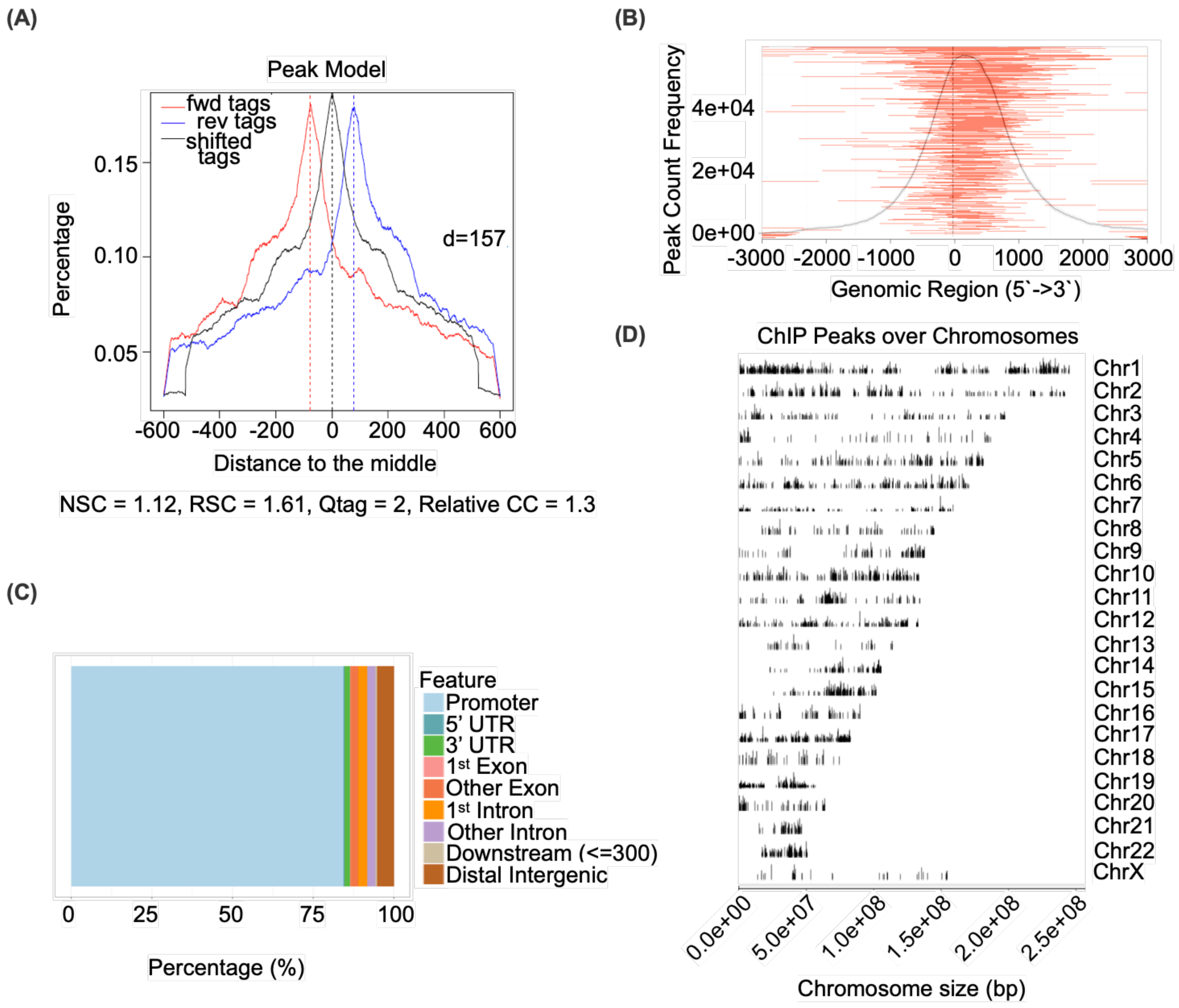

Supplementary Figure S13. Exploratory plots of (in-house) ChIP-seq data analysis. Diagnostic plots clearly revealing the quality of the ChIP-seq peaks called and of the data for reproducibility and replicability (A), the most frequent genomic region where the peaks are called (B), the most frequent genomic features overlapping the peaks called (C), and the distribution of the peaks over chromosomes (D).

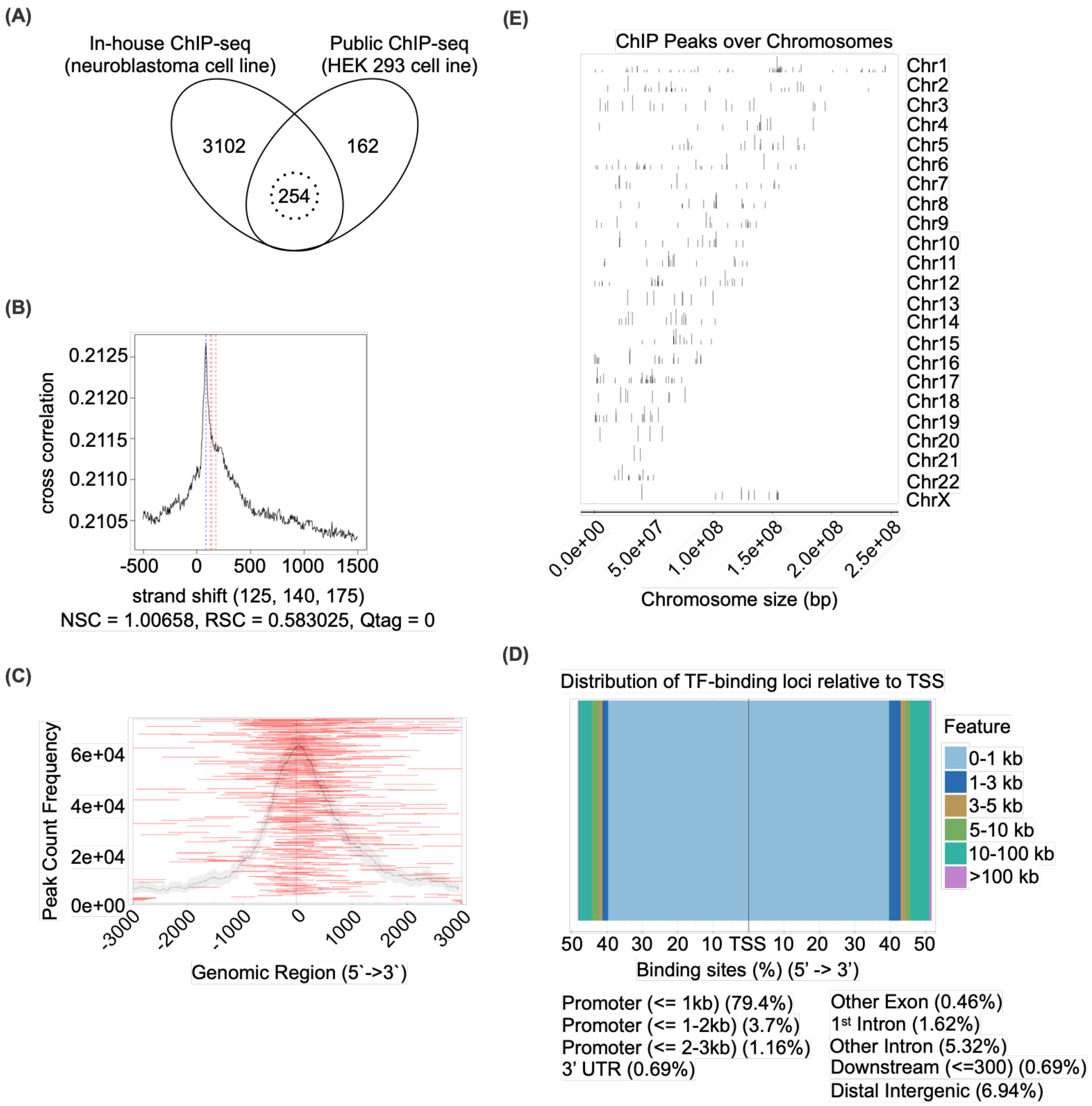

Supplementary Figure S14. Exploratory plots of (public) ChIP-seq data analysis. (A) The number of shared genes between two PARIS ChIP-seq experiments is 254. Diagnostic plots revealing the quality of the ChIP-seq peaks called and of the data for reproducibility and replicability (B), the most frequent genomic region where the peaks are called (C), the most frequent genomic features overlapping the peaks called (D), and the distribution of the peaks over chromosomes (E).

(A)

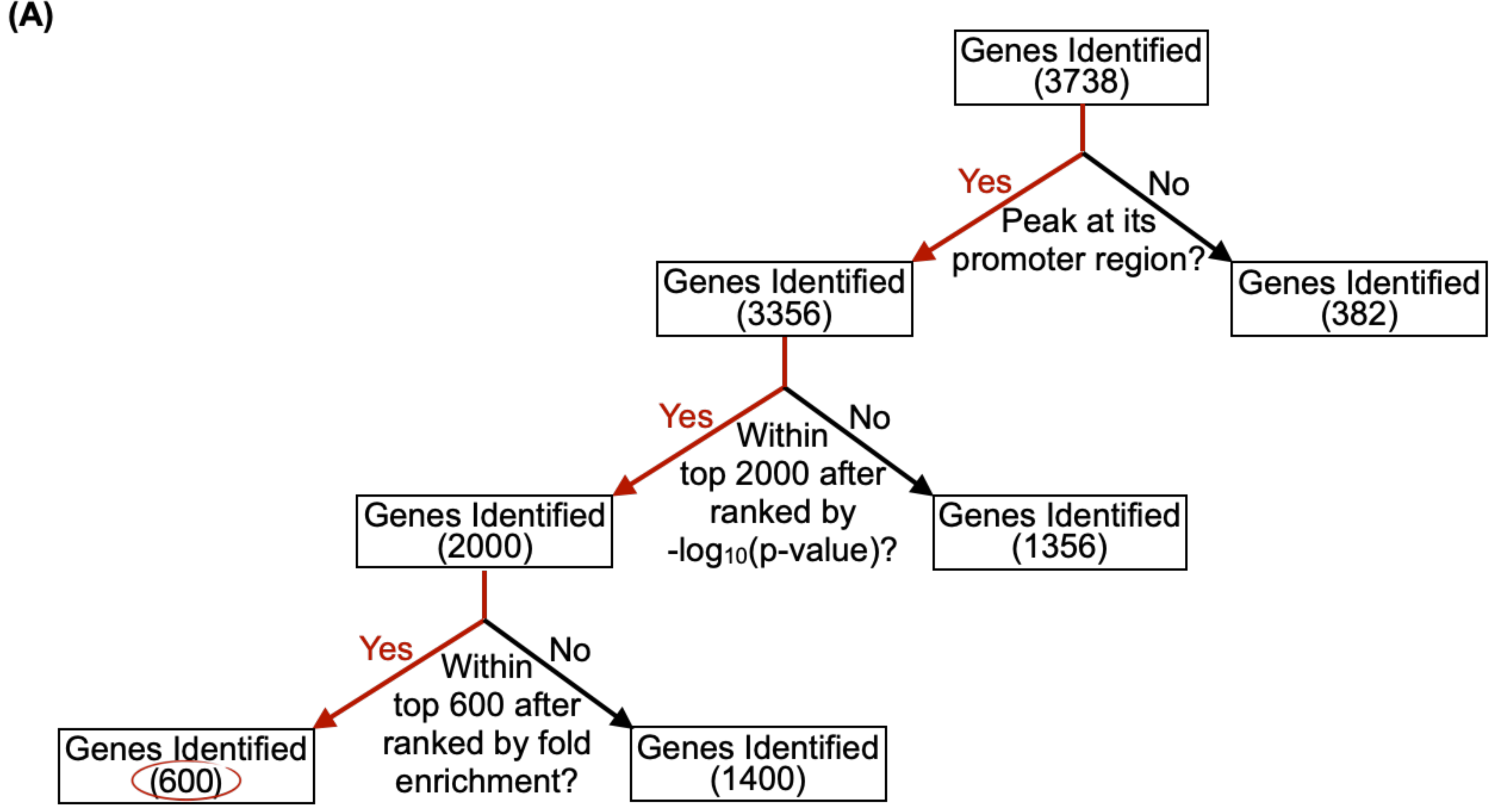

(B)

ENRICHR January 7th, 2020

Rank	Metabolic Pathways	P-value	Adjusted p-value	Odds Ratio
1	Role of PPAR-gamma Coactivators in Obesity and Thermogenesis Homo sapiens h ppargPathway	0.001915	0.1513	11.22
2	Acetylation and Deacetylation of RelA in Nucleus Homo sapiens h RELAPathway	0.008296	0.3933	13.47
3	Oxidative Stress Induced Gene Expression Via Nrf2 Homo sapiens h arenrf2Pathway	0.001691	0.4008	7.48
4	METS affect on Macrophage Differentiation Homo sapiens h etsPathway	0.001691	0.2004	7.48
5	TGF beta signaling pathway Homo sapiens h tgfbPathway	0.002554	0.1514	6.73
6	Signal Dependent Regulation of Myogenesis by Corepressor MITR Homo sapiens h MITRPathway	0.02189	0.4717	8.42
7	Bone Remodeling Homo sapiens h ranklPathway	0.01094	0.3703	6.31
8	Cyclin E Destruction Pathway Homo sapiens h fbw7Pathway	0.02760	0.4673	7.48
9	Regulation of Splicing through Sam68 Homo sapiens h sam68Pathway	0.03384	0.5012	6.73
10	E2F1 Destruction Pathway Homo sapiens h skp2e2fPathway	0.03384	0.4717	6.73

(C)

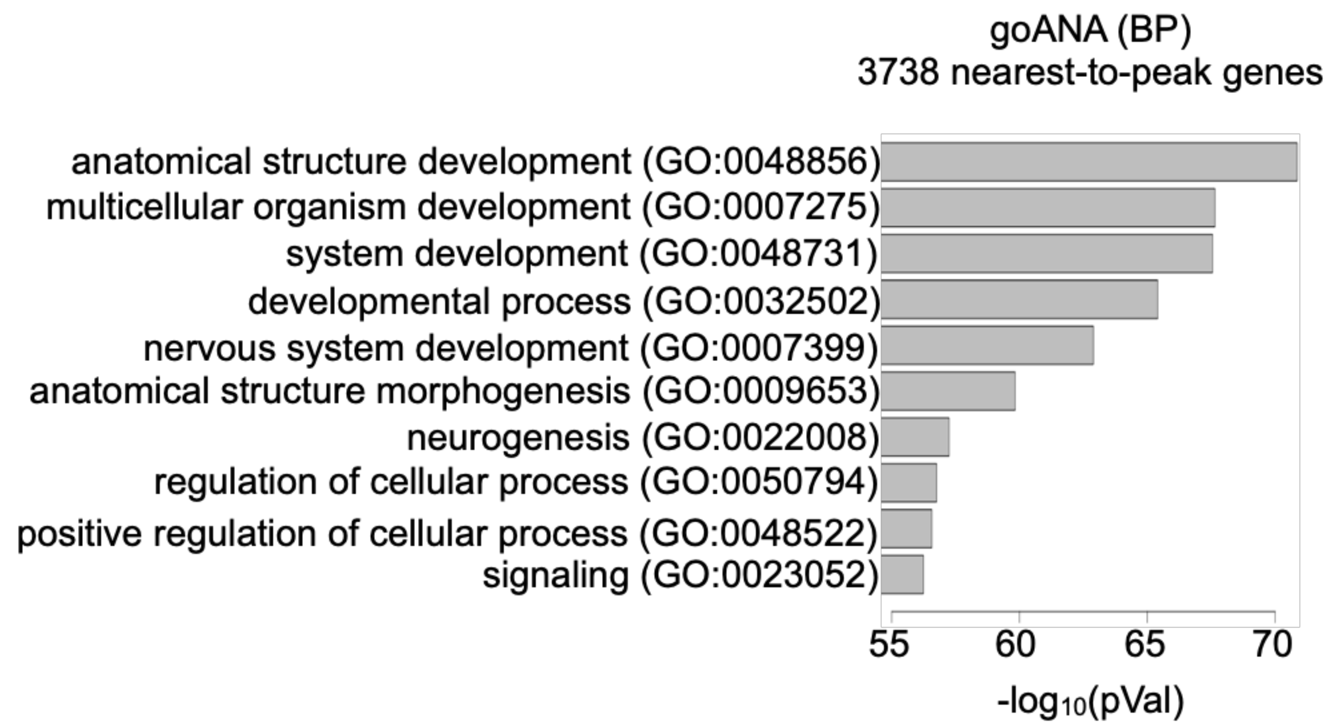

Supplementary Figure S15. BioCarta metabolic pathway analysis (v2016) of top 600 nearest-to-peak genes obtained from ChIP-seq results. (A) The decision tree made to rank, based on peak calling p-value and then on fold enrichment, about 3400 genes to obtain a list of top 600 genes with a ChIP-seq peak overlapping exclusively the promoter region. (B) The top 600 genes were used for BioCarta Metabolic Pathway Analysis, confirming TRAP-seq results, we observed that PPARγ Pathway appears as the most significant pathway enriched in the final list of peak-annotated genes while NRF2 Pathway ranked third. (C) Nervous system-associated GO terms appear enriched in the peak-annotated genes.

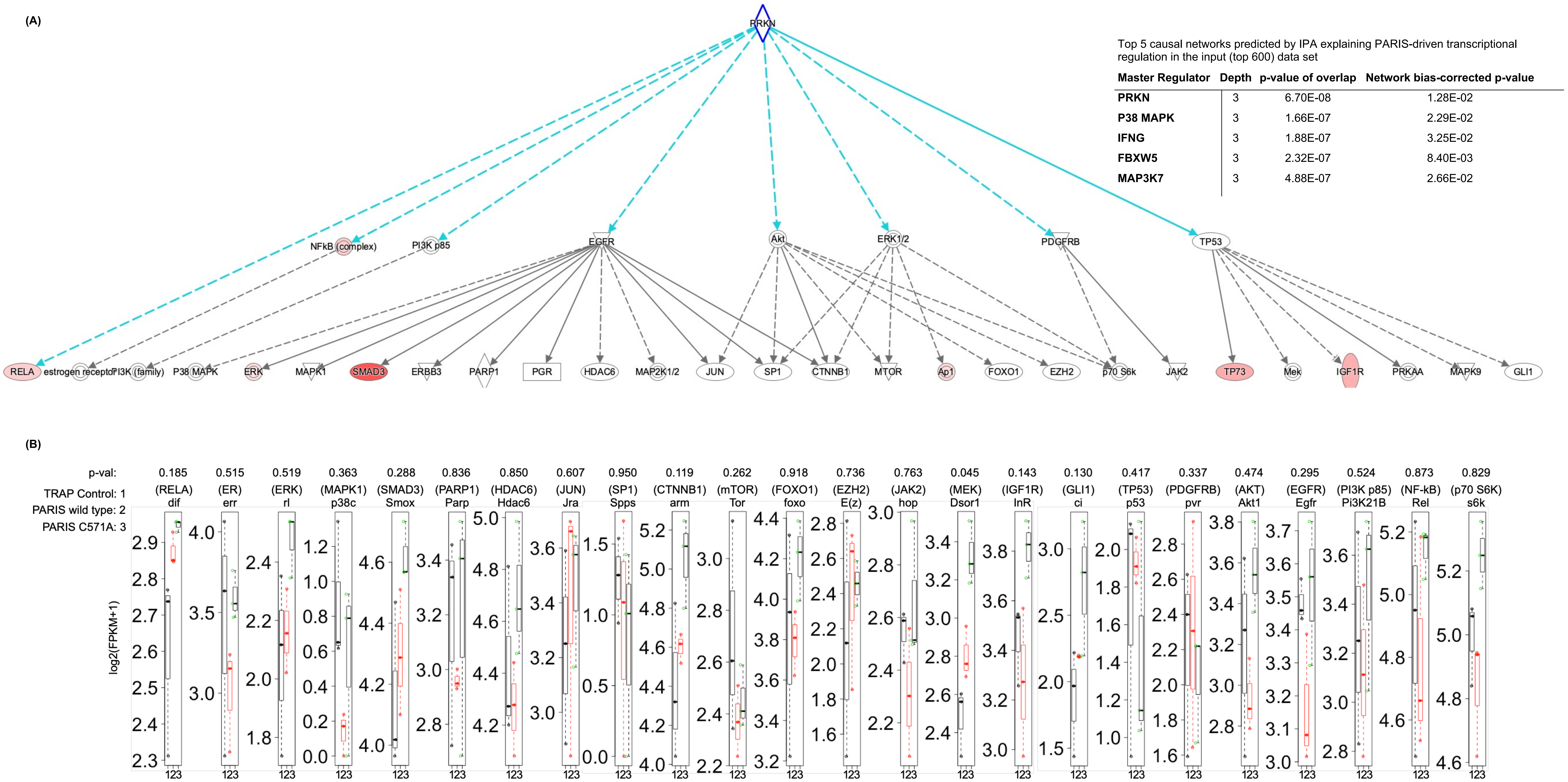

Top 5 causal networks predicted by IPA explaining PARIS-driven transcriptional regulation in the input (top 600) data set

Master Regulator	Depth	p-value of overlap	Network bias-corrected p-value
PRKN	3	6.70E-08	1.28E-02
P38 MAPK	3	1.66E-07	2.29E-02
IFNG	3	1.88E-07	3.25E-02
FBXW5	3	2.32E-07	8.40E-03
MAP3K7	3	4.88E-07	2.66E-02

Supplementary Figure S16. PRKN networks enriched after CNA. (A) The Causal Network Analysis by IPA confirmed that Parkin is the top-most component of the regulatory network associated with the expression patterns observed in the top-ranked 600 peak-annotated genes described in Supplementary Figure S15. All nodes in (A) were quantitatively evaluated in (B). Top 5 causal networks explaining PARIS-driven transcriptional regulation in the same 600 peak-annotated genes are also given.

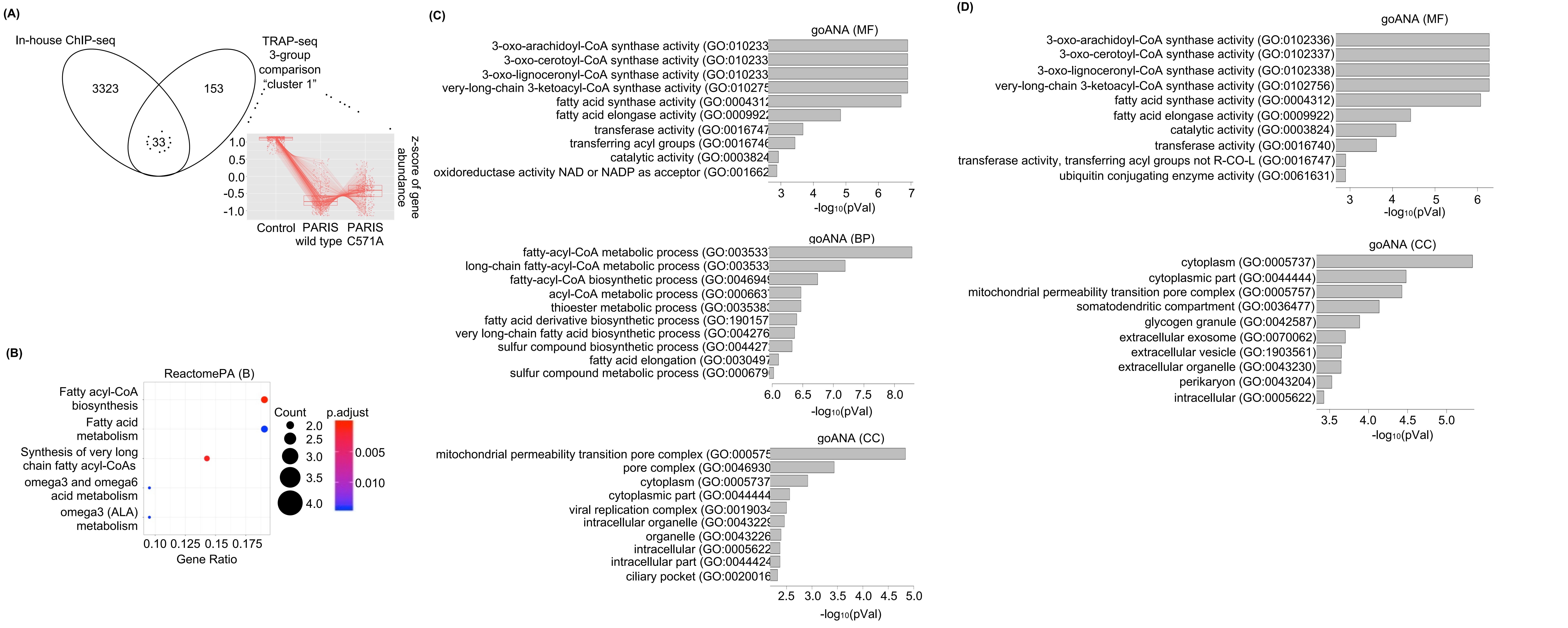

Supplementary Figure S17. Functional enrichment of the Intersection represented by TRAP-seq 3-group comparison “cluster 1” OR downregulated genes from “TRAP Control VS. PARIS wild type” pairwise comparison AND all peak-annotated genes identified from our in-house ChIP-seq dataset. (A) The number of shared genes between TRAP-seq 3-group comparison “cluster 1” and all peak-annotated genes identified from our in-house ChIP-seq dataset is 33. (B) Lipid metabolism associated terms such as fatty acid elongation and acyl-CoA biosynthesis appear as the key module explaining the functional trend in the Reactome Pathway Analysis. PPARγ is a well-known master regulator of lipid metabolism in cell. (C) Similar results were obtained from gene ontology analysis. (D) Functional enrichment results of the shared genes between “TRAP Control VS. PARIS wild type” pairwise comparison and all peak-annotated genes identified from our in-house ChIP-seq dataset yield almost identical results observed in (B).

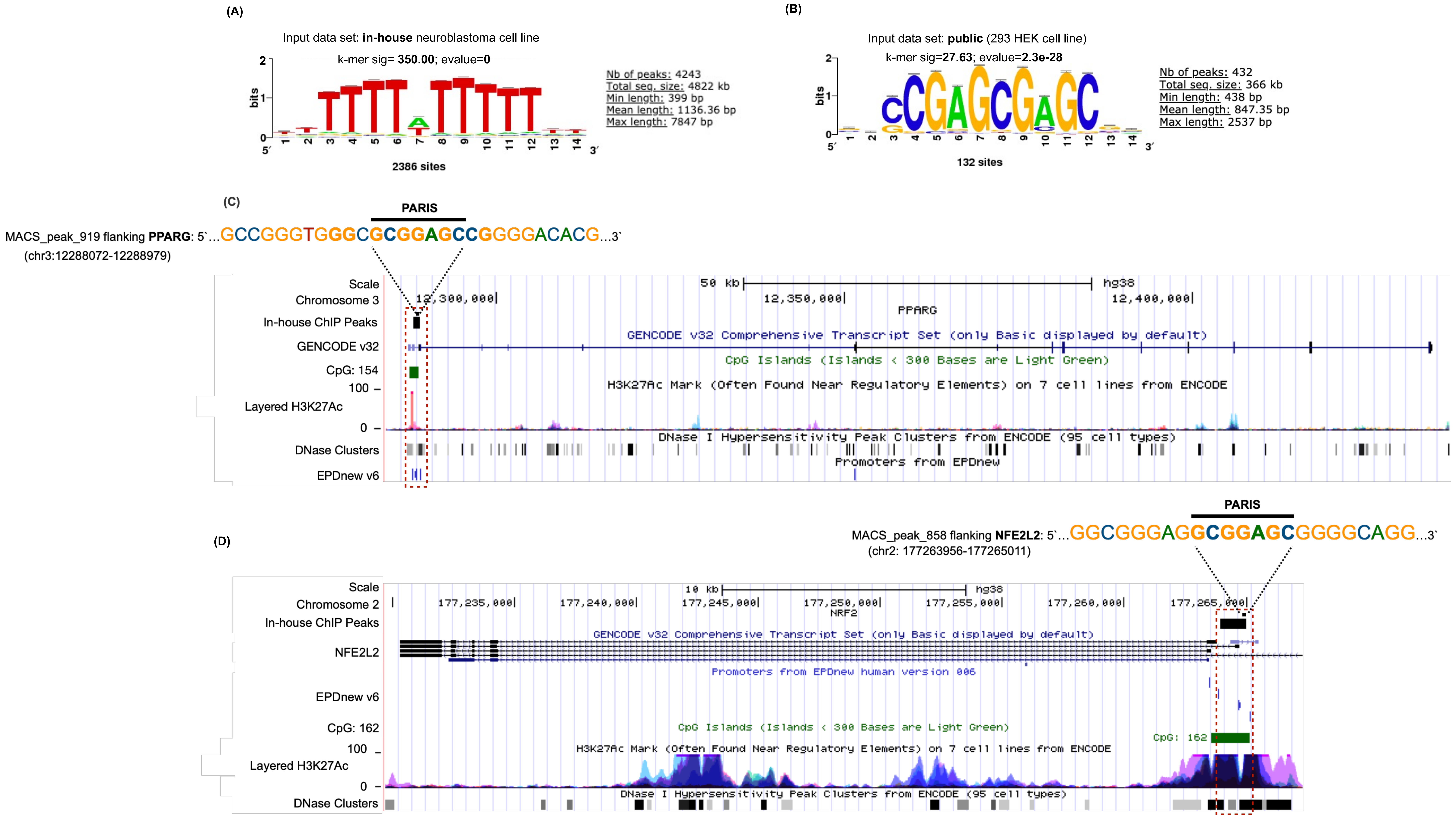

Supplementary Figure S18. PARIS binding motif analysis. (A) We replicated a similar version of the suggested PARIS binding motif published by our group in 2011, which was identified using another popular method back then. (B) The most frequently observed PARIS binding motif in this study was confirmed using two independent ChIP-seq data sets by two technically different motif finder algorithms. (C) PPARγ promoter region contains binding motif for PARIS. Graphical representation of PARIS binding to the PPARγ promoter region predicted by EPDnew v6 feature of the UCSC Genome Browser is also given. (D) NFE2L2 (i.e., NRF2) promoter region contains binding motif for PARIS, implying a direct regulatory effect of PARIS on these target genes.

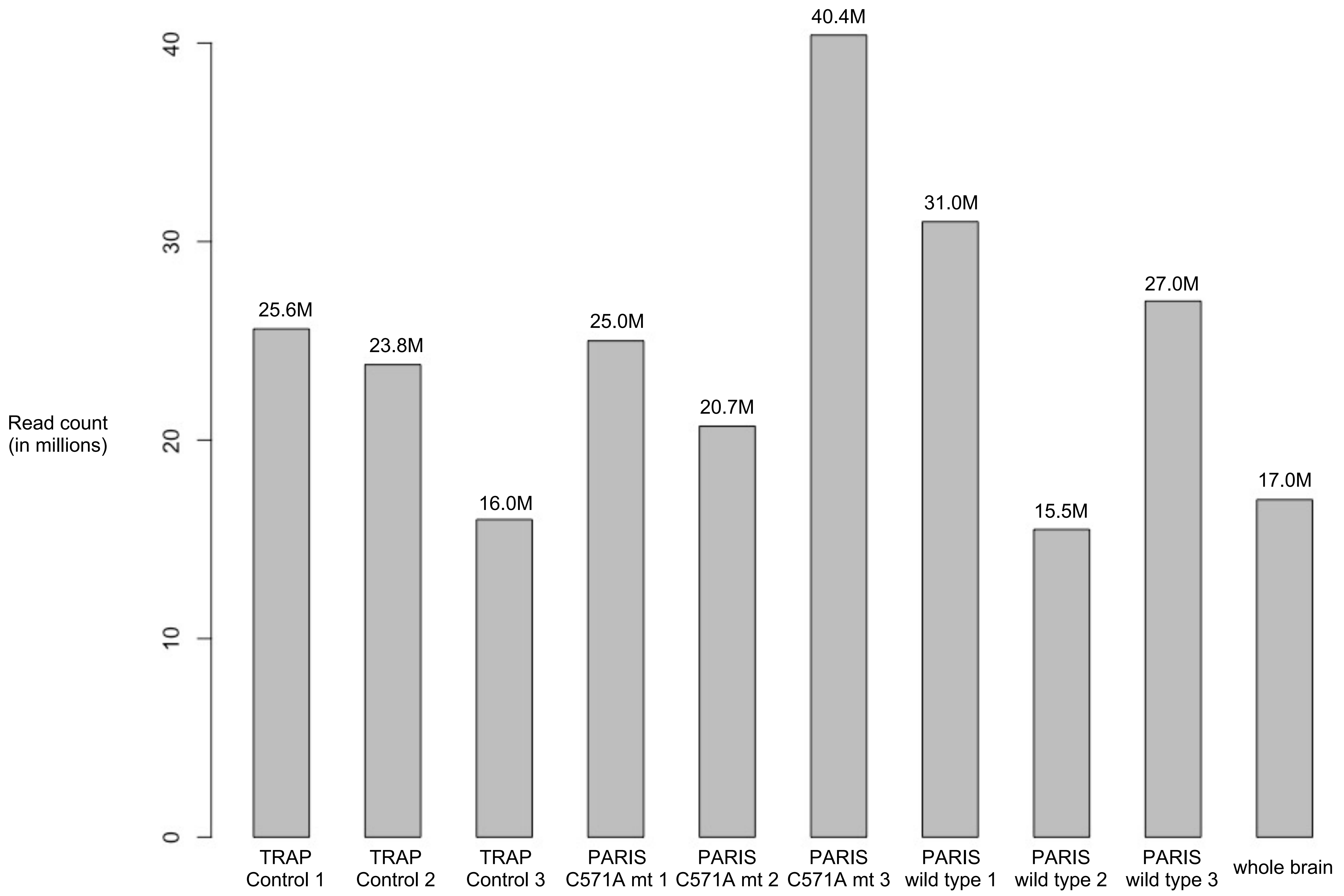

	TRAP Control 1	TRAP Control 2	TRAP Control 3	PARIS C571A mt 1	PARIS C571A mt 2	PARIS C571A mt 3	PARIS wild type 1	PARIS wild type 2	PARIS wild type 3	whole brain
Overall Alignment Rates (%):	97.74	97.51	98.08	98.05	97.97	98.26	98.83	98.67	98.82	98.85
Multimapped Reads (%):	53.71	47.99	53.46	41.47	42.89	43.15	59.63	51.92	54.26	12.89
***rRNA-based gDNA Alignment Rates (%):	51.29	45.07	50.91	38.41	39.65	40.72	59.55	50.06	52.99	3.01

***Sequence Identity of the rRNA Genes in D. melanogaster: 95% of the genomic reads representing rRNA genes in fruit fly genome (GCF_000001215.4 assembly) have min 93.64% sequence identity

Supplementary Figure S19. Distribution of read counts across samples. The read depths and overall alignment rates are significantly high across the samples used within the scope of this work. It is also shown that the high percentage of multimapped reads is a direct result of read alignments to the ribosomal RNA genes in the fly genome with a well over 90% sequence identity.

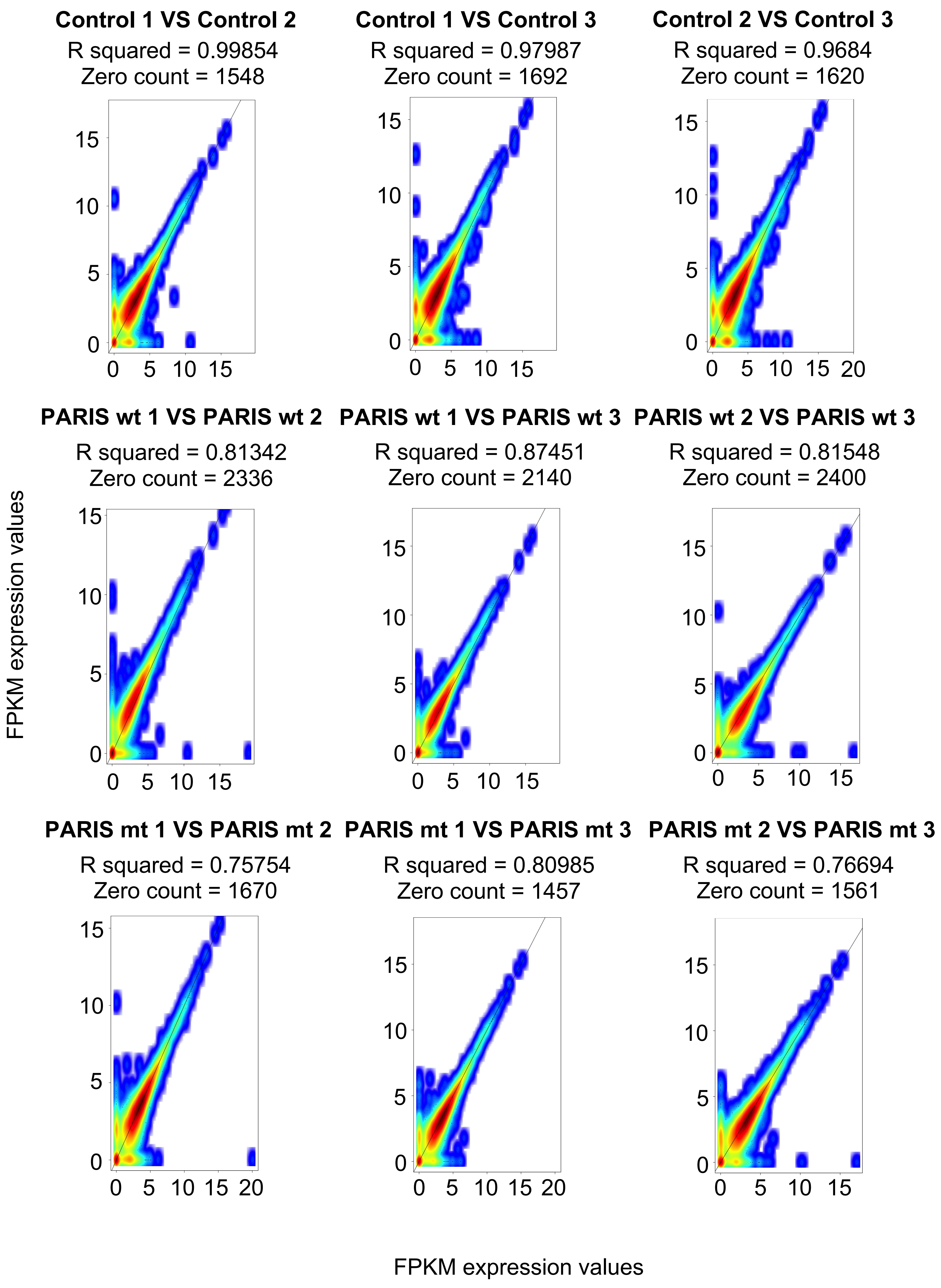

Supplementary Figure S20. Reproducible analysis of all biological replicates in each group. High degree of correlation among the biological replicates in each group has been confirmed.

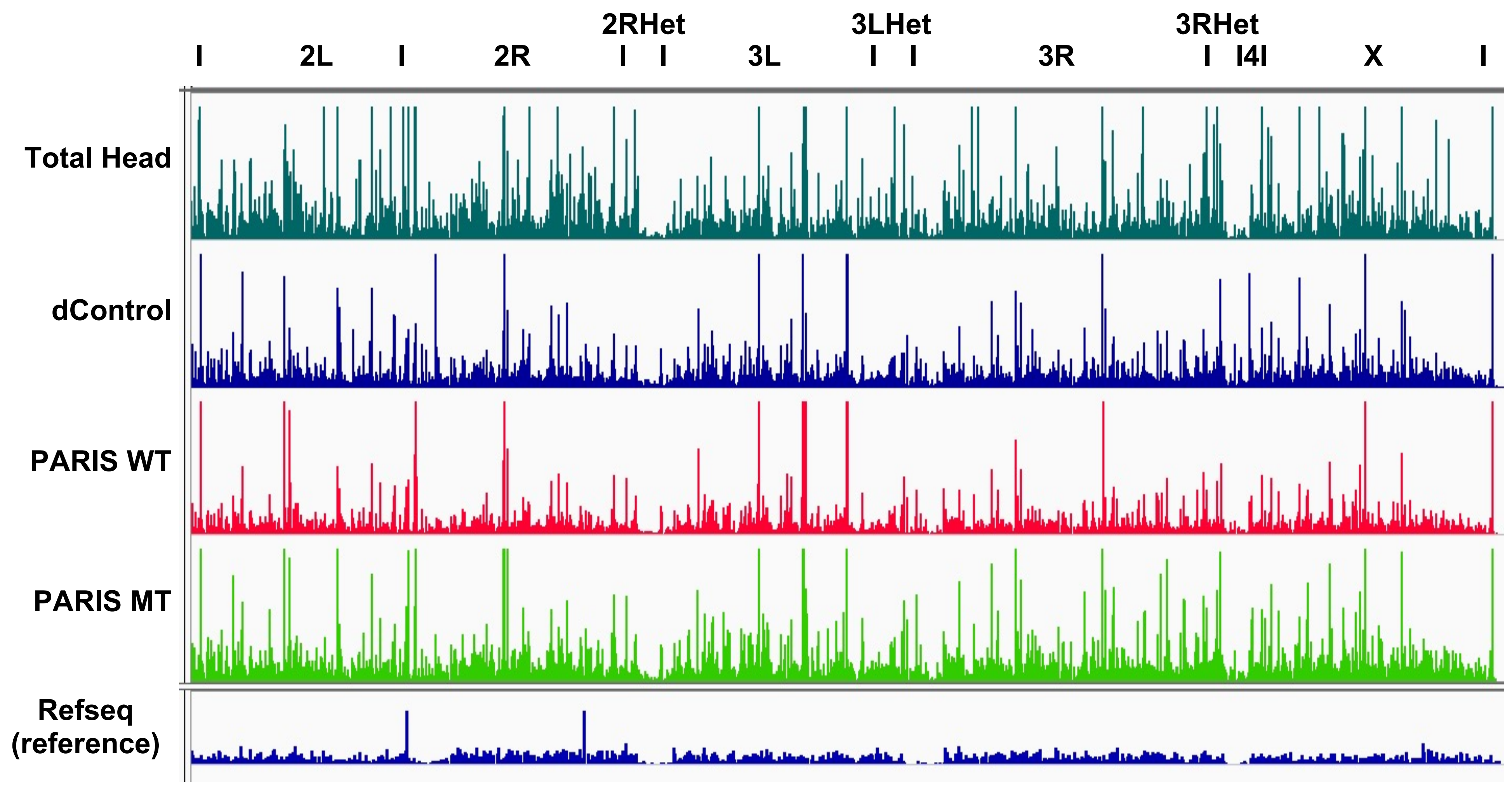

Supplementary Figure S21. Genome-wide distribution of reads across sample groups. TRAP protocol does not have a global impact on gene expression in Drosophila.

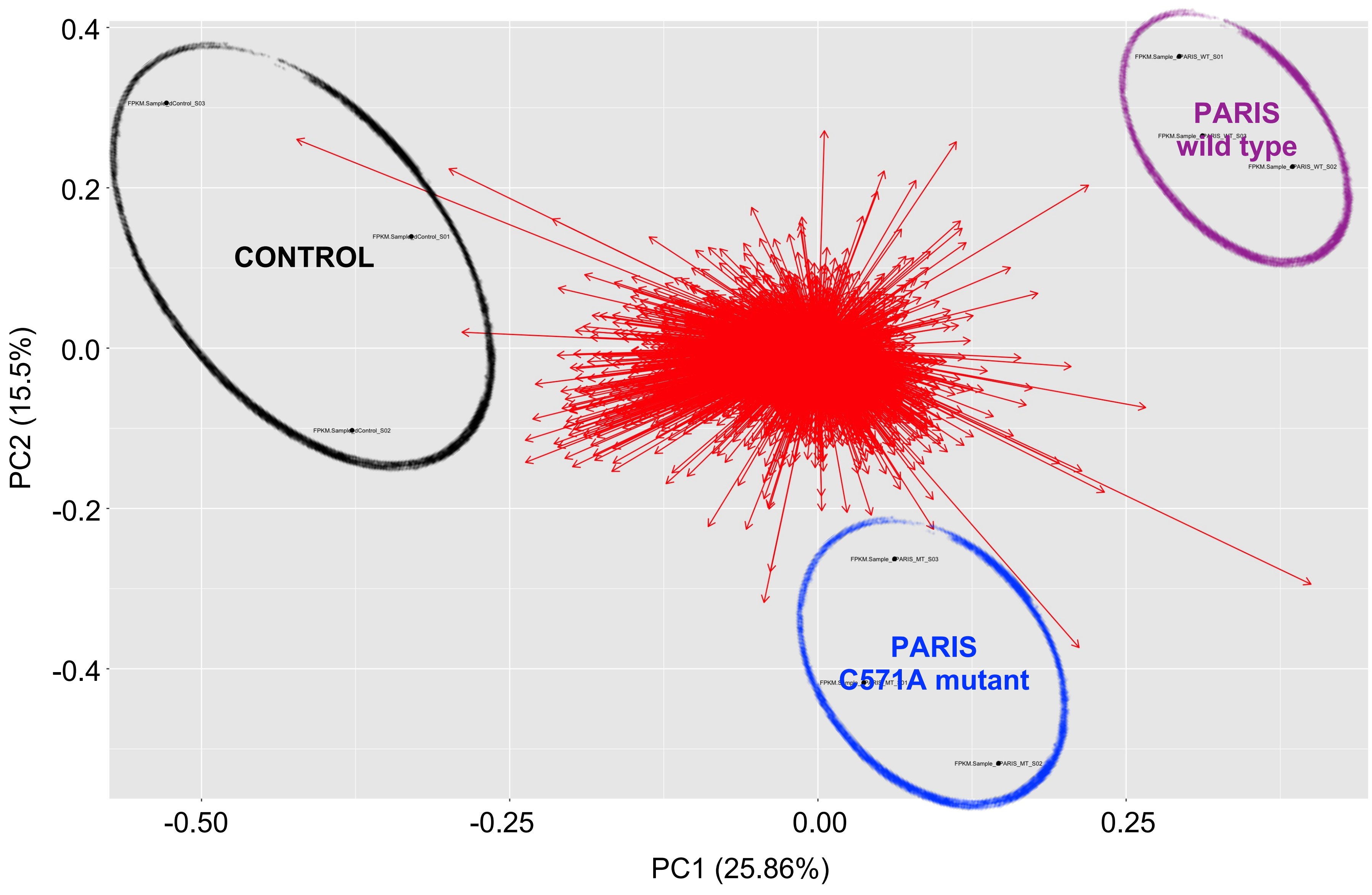

Supplementary Figure S22. Principle component aanalysis of the filtered data set. The replicates of each sample group cluster together, generating three distinct groups along the PC1 and PC2.

TRAP Control VS. PARIS wild type VS. PARIS C571A mutant

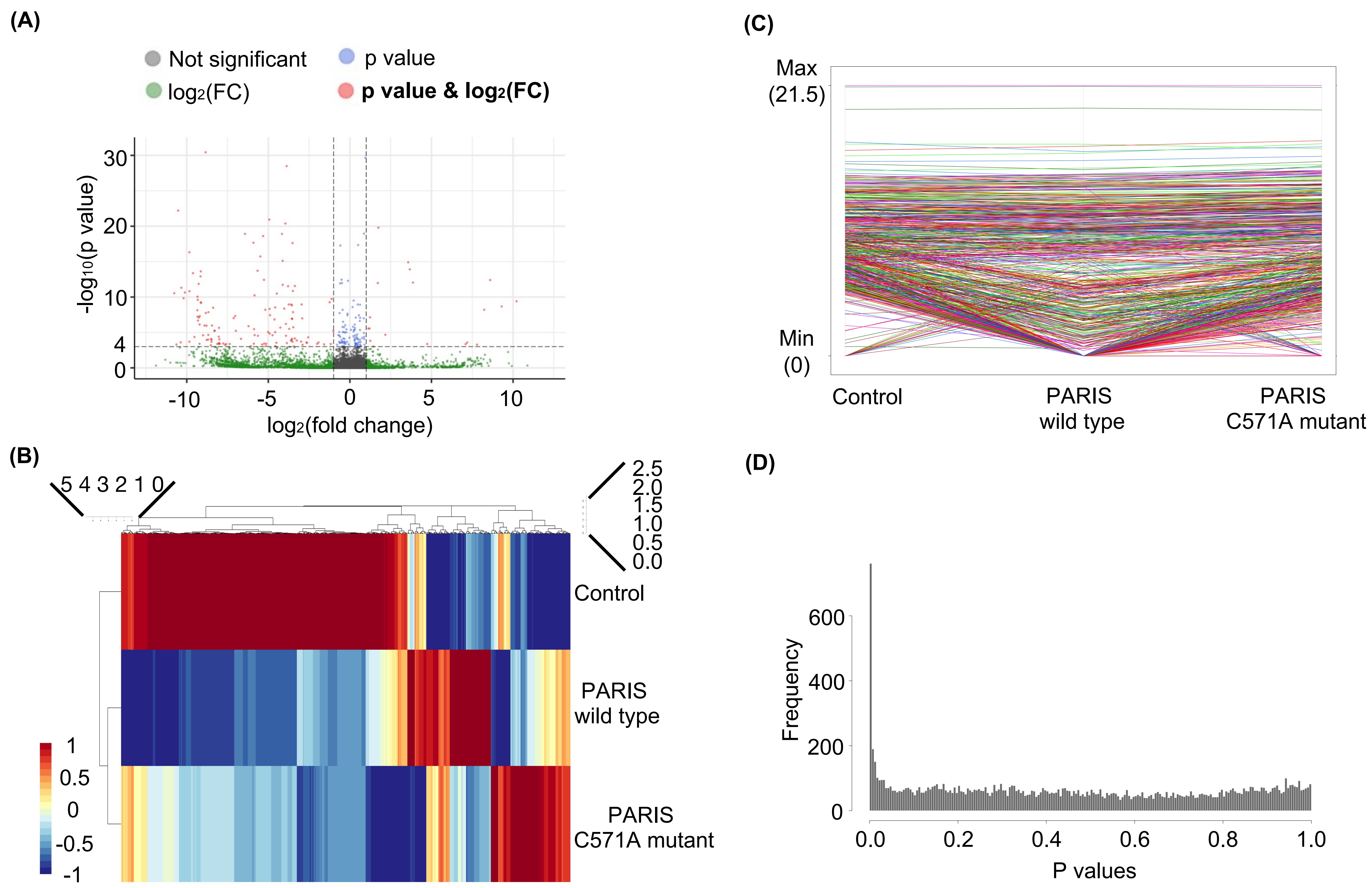

Supplementary Figure S23. Exploratory plots depicting changes in expression levels of the count data from 3-group comparison. (A) Volcano plot of expression data reveals a clear trend of downregulation induced by PARIS. Red dots represent significant genes with high expression fold change. (B) Distinct clusters of genes sharing common expression patterns are observable in a heatmap. (C) A parallel coordinate plot showing changes in expression of each gene across different sample groups. Each line represents expression profile of a gene in the input dataset. (D) A p-value histogram confirming the expected uniform distribution of null p-values with a peak close to “0”, where the alternative hypotheses reside.

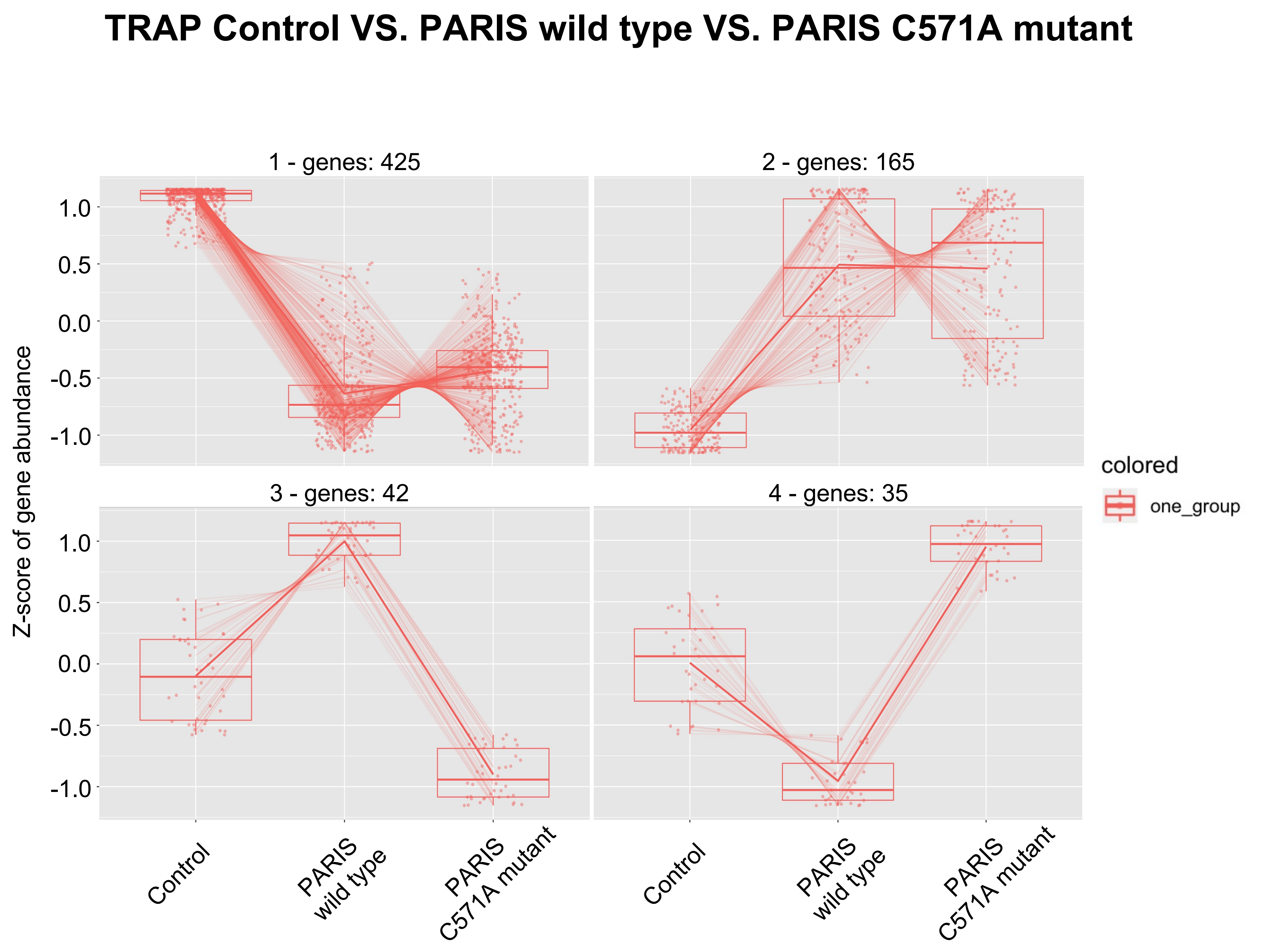

Supplementary Figure S24. Gene clusters exhibiting particular patterns across samples. 3-group comparison yields 4 different patterns of significant expression changes.

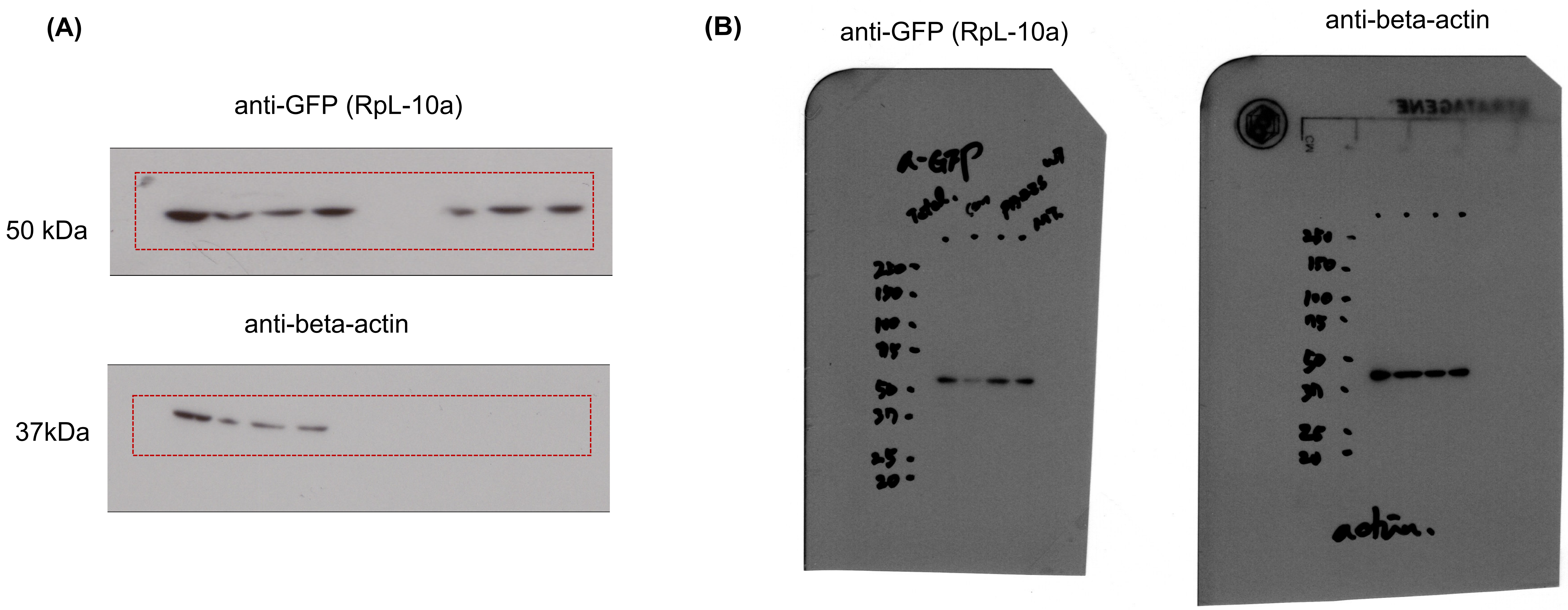

Supplementary Figure S25. Western blots with cropped (dotted red lines presented in Fig. 1b) images. Original membranes were cut prior to hybridization with antibodies during blotting (A). To provide specific detection of the target antigen, the full-size immunoblotting results are presented using input samples (B).
